# Design of Mimetic Antibodies Targeting the SARS-CoV-2 Spike Glycoprotein Based on the GB1 Domain: A Molecular Simulation and Experimental Study

**DOI:** 10.1021/acs.biochem.4c00671

**Published:** 2025-03-17

**Authors:** Anderson A. E Santo, Aline Reis, Anderson A. Pinheiro, Paulo I. da Costa, Gustavo T. Feliciano

**Affiliations:** † Institute of Chemistry, São Paulo State University, Araraquara, SP 14800-900, Brazil; ‡ School of Pharmaceutical Sciences, São Paulo State University, Araraquara, SP 14801-360, Brazil

## Abstract

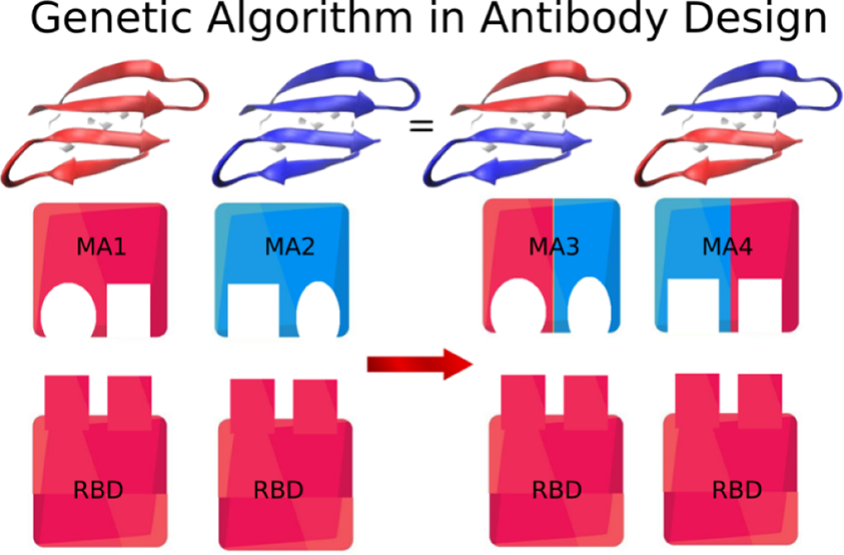

In the context of fast and significant technological transformations, it is natural for innovative artificial intelligence (AI) methods to emerge for the design of bioactive molecules. In this study, we demonstrated that the design of mimetic antibodies (MA) can be achieved using a combination of software and algorithms traditionally employed in molecular simulation. This combination, organized as a genetic algorithm (GA), has the potential to address one of the main challenges in the design of bioactive molecules: GA convergence occurs rapidly due to the careful selection of initial populations based on intermolecular interactions at antigenic surfaces. Experimental immunoenzymatic tests prove that the GA successfully optimized the molecular recognition capacity of one of the MA. One of the significant results of this study is the discovery of new structural motifs, which can be designed in an original and innovative way based on the MA structure itself, eliminating the need for preexisting databases. Through the GA developed in this study, we demonstrated the application of a new protocol capable of guiding experimental methods in the development of new bioactive molecules.

## Introduction

The SARS-CoV-2 coronavirus is the etiological agent of COVID-19, and was named due to it is great genomic similarity with the original SARS-CoV virus, now officially known as SARS-CoV-1.^1^ Identified in the city of Wuhan, China, SARS-CoV-2 is an enveloped Betacoronavirus (βCoVs) whose genome is a positive single strand RNA (+ssRNA) molecule approximately 30,000 bases long.^2^ Among the proteins encoded by the virus, there are four structural ones: (1) the nucleocapsid phosphoprotein; (2) membrane glycoprotein (M); (3) the envelope protein (E); and finally, (4) the surface glycoprotein, also known as Spike or S protein, the focus of this work.

Spike, in addition to being a transmembrane structural protein, is a class I fusion protein, whose function is to allow SARS-CoV-2 to enter its host cells. This glycoprotein is divided into two subunits (S1 and S2), each with its own function.^3^ It is in the S1 subunit that the receptor-binding domain (RBD) is found, the region responsible for binding the virus to the cell membrane through its receptor, the angiotensin-converting enzyme type 2 (ACE2). The S2 subunit, in the other hand, is involved with the process of fusion of the viral envelope with the cell membrane, and it is at this subunit that the transmembrane domain (TM), the fusion peptide (FP), and the internal fusion peptide (IFP) are found.^4,5^

One of the most efficient ways to combat new viral agents is through the development of antibodies capable of neutralizing viruses and generating an immune response.^6^ From a pharmacodynamic point of view, antibodies are the ideal therapeutic agents due to their high affinity and selectivity, which helps to avoid the common side effects common of other medications. However, antibody design is one of the most challenging tasks due to the large variability of the CDRs (complementarity-determining regions).^7^ Currently existing software, such as Rosetta,^8^ is primarily intended to improve existing antibodies by increasing their affinity for the antigen.

Due to its essential function on the virus replication cycle, the RBD emerged as target of great interest for the development of antibody-based therapies for the treatment of SARS-CoV-2.^9^ However, finding, characterizing, producing and purifying specific antibodies is one of the most challenging tasks, resulting in extremely high production costs.^10^ In this scenario, the development of mimetic antibodies (MA) is justified. MA are peptides capable of performing the molecular recognition function of antibodies. MA technology has great potential for applications in the healthcare sector, whether for use in biosensors or in the development of new biopharmaceutical drugs.^11^ The main advantage of these molecules is their lower production cost compared to monoclonal antibodies, as MA can be expressed by bacteria in simple cloning vectors, making them much more accessible.

For this purpose, scientists have been exploring innovative approaches in the fields of artificial intelligence (AI),^12−14^ aiming to accelerate the development of bioactive molecules and address today’s major challenges. However, this type of project has its own limitations, such as dependence on reliable databases,^15^ necessary for machine learning and neural networks. Furthermore, in the case of computationally designed molecules, there is not always a guarantee of an available synthetic route or feasible accessibility. As a result, many researchers choose to work with peptides due to their relative ease of synthesis.

Our group has been continuously developing a new method for MA design using the streptococcal G protein GB1 domain peptide^16^ as a structural basis, which consists of only 56 amino acids. Due to its high structural stability, this peptide is highly resilient to amino acid modifications. Thus, it serves as an ideal candidate for a basis in MA design, where surface residues can be modified. This new method is characterized by the integration of traditional protein molecular dynamics (MD) techniques^17−19^ and free binding free energy (Δ*G*_bind_) calculation using the MM-GBSA method.^20,21^ The protocol is structured in the form of a genetic algorithm (GA),^16^ capable of optimizing the development of new bioactive molecules.

**Figure 1:**
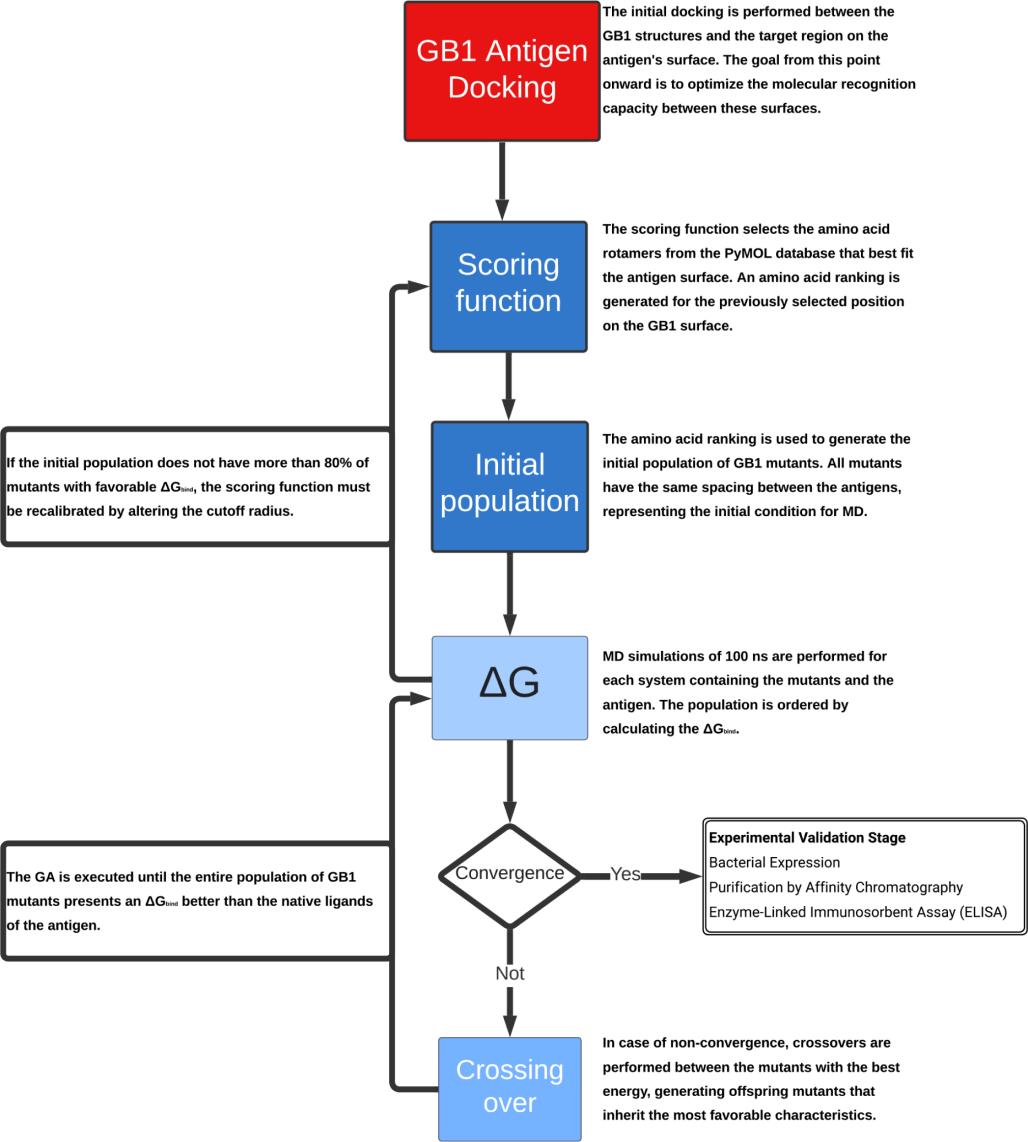
Project flowchart showing all steps of the GA protocol.

GAs are a class of optimization algorithms within the field of AI,^14^ inspired by Darwin’s theory of evolution,^22,23^ and are used to find solutions to multidimensional problems. In our project, shown in Figure 1, this optimization is based on an initial population generated by a simple scoring function, capable of selecting the residues that best interact with the surface of the target antigen,^24,25^ allowing faster convergence. It is important to highlight that, as suggested by Santo and Feliciano (2021), GAs are an innovative tool particularly relevant for the design of biomolecules^26^ through AI. This is primarily because MD-based GA considers interactions between distant residues within the ligand itself,^27^ unlike other approaches, such as traditional machine learning algorithms, which primarily rely on labeled data for classification.^15^

In this work, we propose the design of MA targeting the SARS-CoV-2 RBD using the GB1 domain as the structural scaffold, and the experimental validation through structural analysis, protein expression and neutralization tests to confirm the intended properties. The proposal outlined in this study aims to reconsider the applicability of widely used computational techniques in academic research, such as molecular dynamics simulations and artificial intelligence algorithms, redefining them not only as tools for analyzing more intricate biological systems but also as active agents in their transformation and improvement. Here, we demonstrate that such transformation can be achieved through the reprogramming of biological functions, thus facilitating the creation of new biotechnological products.

## Materials and Methods

### MD Parameters

All MD simulations were performed on a GPU using pmemd.cuda implemented in Amber 18.^28^ Each system was solvated with the TIP3P water model^29^ in a simulation cubic box, with a 1 nm distance between the system and the box edge. The particle mesh Ewald method was used to handle long-range nonbonded interactions.^30^ For short-range interactions, a standard Amber cutoff radius of 8 Å was employed.^31^ The classical force field considers a fixed protonation state of the protein at pH 7. Each system underwent an energy minimization process with the steepest descent algorithm.^32^ The system temperature was raised to 300 K using a Langevin dynamic thermostat.^33^ The pressure was maintained at 1 bar using the Berendsen barostat^34,35^ Amber default settings. All MD simulations were conducted in two steps. In the first step, a simulation was performed for a period of 90 ns, during which temperature, pressure, and the positions of the first and last frames were saved. The second step of the simulation was then continued from 90 to 100 ns, where 5000 frames were saved.^16^ The binding free energy was calculated using the MMPBSA.py script from AmberTools16, using a salt concentration of 0.1 and the dielectric constant of water. In this project, we utilized a machine equipped with an Intel Xeon Gold 6130 processor and an NVIDIA Geforce GTX 1080 graphics card. The choices made for the MD simulations are justified by our primary goal in GA, which is to optimize Δ*G*_bind_. For this, computational efficiency is prioritized over building more complex and realistic models, which would be more computationally expensive.

### MM-GBSA

The Δ*G*_bind_ is a thermodynamic quantity that allows estimating the affinity between biomolecules. Among the computational methods for estimating the Δ*G*_bind_, the MM-GBSA occupies a prominent place.^31,36^ This method uses the fact that the Δ*G*_bind_ is a state function to make an alternative thermodynamic path, which simplifies the calculations, as we can see in eq 1:
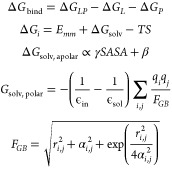
1

The Δ*G*_bind_ in MM-GBSA is decomposed into molecular mechanical energy components *E*_*MM*_, solvation energy Δ*G*_*solv*_ and entropic component energy *T*Δ*S*. The *E*_*MM*_ can be obtained through the force field used in molecular dynamics, Δ*G*_*solv*_ can be decomposed into Delta Δ*G*_*solv,apolar*_, which is nothing more than the work required to generate the cavity in the solvent where the system is located and Δ*G*_*solv,polar*_, which can be interpreted as the potential resulting from the shielding that the ions in the medium exert on the surface of the system.^21^ In MM-GBSA, the water reorganization entropy is encapsulated within the polar solvation term. Terms of vibrational, rotational, and translational entropy must be calculated by other means.

### MA Expression and Reactivity Evaluation

*Escherichia coli* BL21 (DE3) Rosetta cells, which had been transformed with the expression plasmids pET-28a/GB1-REF and pET-28a/GB1–121, were cultured in LB broth until reaching an optical density (OD) of 0.4 at 600 nm. Protein expression was initiated by adding isopropyl β-D-1-thiogalactopyranoside (IPTG) to a final concentration of 1 mM and incubating at 37 ^◦^C for 4 h. After the induction period, the cells were collected by centrifugation at 4000 rpm for 10 min.

The collected cells were resuspended in lysis buffer (containing NaCl 0.01 M, NaH_2_PO_4_ 0.05 M, PMSF 0.001 M, Tris-HCl 0.01 M, EDTA 0.001 M, DTT 5 mM, and 2% Triton X-100) and sonicated for 10 min to lyse the cells. Following sonication, the lysate was centrifuged at 12,000 rpm for 10 min to separate the components. For purification, the recombinant proteins were subjected to affinity chromatography using a HisTrap High Performance 1 mL Column (Cytiva 17–5247–01). The desired fractions were eluted with an Elution buffer (containing NaH_2_PO_4_ 20 mM, NaCl 500 mM, and Imidazole 500 mM). A chromatographic profile was obtained using the Micro BCA method to assess protein levels.

The fractions containing the eluted proteins were combined and then dialyzed using a 3 kDa membrane to remove excess imidazole and facilitate the renaturation of the MAs by transitioning the solvent to PBS. The expression was confirmed by immunodot onto a nitrocellulose membrane. The reactivity of the GB1-REF and GB1–121 was evaluated by competitive ELISA using the cPass SARS-CoV-2 Neutralization Antibody Detection Kit (GenScript). The experimental protocols are shown in more detail in the SI.

## Results and Discussion

### Genetic Algorithm Protocol

**Figure 2:**
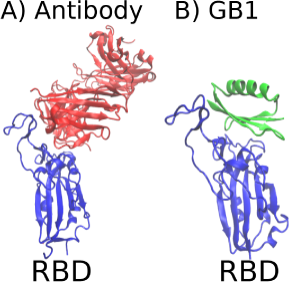
Illustration of the region of the RBD domain (Blue) neutralized by antibodies. A) Structure of the Fab fragments of the B38 antibody (Red) neutralizing the ACE2 recognition region. B) Structure of the GB1 domain (Green) in the molecular recognition optimization region.

To construct the theoretical model of the system, we utilized the structure of the RBD domain of the SARS-CoV-2 spike protein (PDB ID: 7BZ5)^37^ and the structure of the GB1 domain of the streptococcal G protein (PDB ID: 3GB1).^38^ In Figure 2A, we can see how the Fab fragment of the B38 antibody^37^ binds to the ACE2 recognition region, blocking the virus’s entry mechanism into the cell. This region is the most important of the RBD because, in addition to blocking the molecular recognition function, it allows for the comparison of the Δ*G*_bind_ of the B38 antibody. Therefore, we chose to optimize the molecular recognition capability of GB1 in this region. As described by Santo and Feliciano (2021), we used the UCSF Chimera software^39^ to position the GB1 peptide in the same binding region as B38, a region where we want to optimize molecular recognition, as can be seen in Figure 2B:

Due to the large size of antibodies, there are many binding microdomains capable of maintaining molecular recognition capability. However, this flexibility is limited by the small size of GB1. Therefore, we chose to use the average Δ*G*_bind_ between the two Fab fragments of the B38 antibody, which are closer in size to GB1. In this case, we obtain the Δ*G*_bind_ of −50.23 kcal.mol^–1^ used as the convergence criterion. The structures of the RBD antigen and the GB1 peptide shown in Figure 2B were used as the initial condition of the GA.

### Scoring Function

The scoring function, based on empirical parameters, is explained in detail in the previous work of Santo and Feliciano (2021). Its purpose is to determine which residues best fit each position of the GB1 template, allowing us to generate a nonrandom initial population. The code that implements this functionality is shown in the’Data and Software Availability’ section of this paper and was applied to the system proposed in Figure 2B. This function can be interpreted as a way of positioning the initial population of MA within the limits of a local minimum, which is sought with the GA. The scoring function ranks the residues at each position {2, 4, 6, 8, 13, 15, 17, 19, 42, 44, 46, 49, 51, 53, 55} of GB1. The columns showing the top-ranked amino acid residues at each position for the GB1 with the RBD antigen are presented in Table 1.

**Table 1:** Order of the Highest Ranked Amino Acid Residues at Each Position in the Rotamer Scoring Function

Residues															
Code	2	4	6	8	13	15	17	19	42	44	46	49	51	53	55
SGB1–1	Y	L	W	M	E	A	L	L	Q	W	H	A	G	W	Y
SGB1–2	E	I	Y	T	M	L	Y	I	L	I	W	I	A	R	M
SGB1–3	K	V	H	V	A	V	R	Y	V	Y	Y	L	V	K	K
SGB1–4	W	H	R	I	R	T	K	W	Y	K	Q	S	I	Y	W
SGB1–5	I	M	E	N	K	Y	H	E	N	L	R	K	S	E	L
SGB1–6	R	E	I	Q	W	N	W	R	A	T	E	M	T	I	T
SGB1–7	H	R	L	E	V	D	V	Q	R	Q	M	N	Y	H	N
SGB1–8	Q	Q	Q	R	H	E	T	M	K	D	I	D	N	Q	Q
SGB1–9	M	S	N	L	P	H	E	V	S	V	L	G	E	M	R
SGB1–10	N	K	T	A	L	W	N	S	T	H	N	Q	L	V	I
SGB1–11	T	T	V	Y	I	M	D	T	W	S	A	H	P	T	H
SGB1–12	S	N	S	H	T	I	A	G	P	N	P	P	R	S	S

The generation of the initial population using a simple scoring function is not intended to produce individuals with affinity for the molecular recognition region. Instead, its primary purpose is to address challenges associated with the random generation of an initial population, such as the placement of individuals in highly unfavorable regions. This approach demonstrates the effectiveness of the scoring function, defined by empirical parameters, as a reliable method for initializing populations. Moreover, the results suggest that the antigen’s surface region exhibits diverse interaction types, facilitating the differentiation of residues. This information is subsequently utilized to generate an initial population for application in the GA, avoiding the need to test all 20^15^ possible amino acid combinations.

### Genetic Algorithm

**Figure 3:**
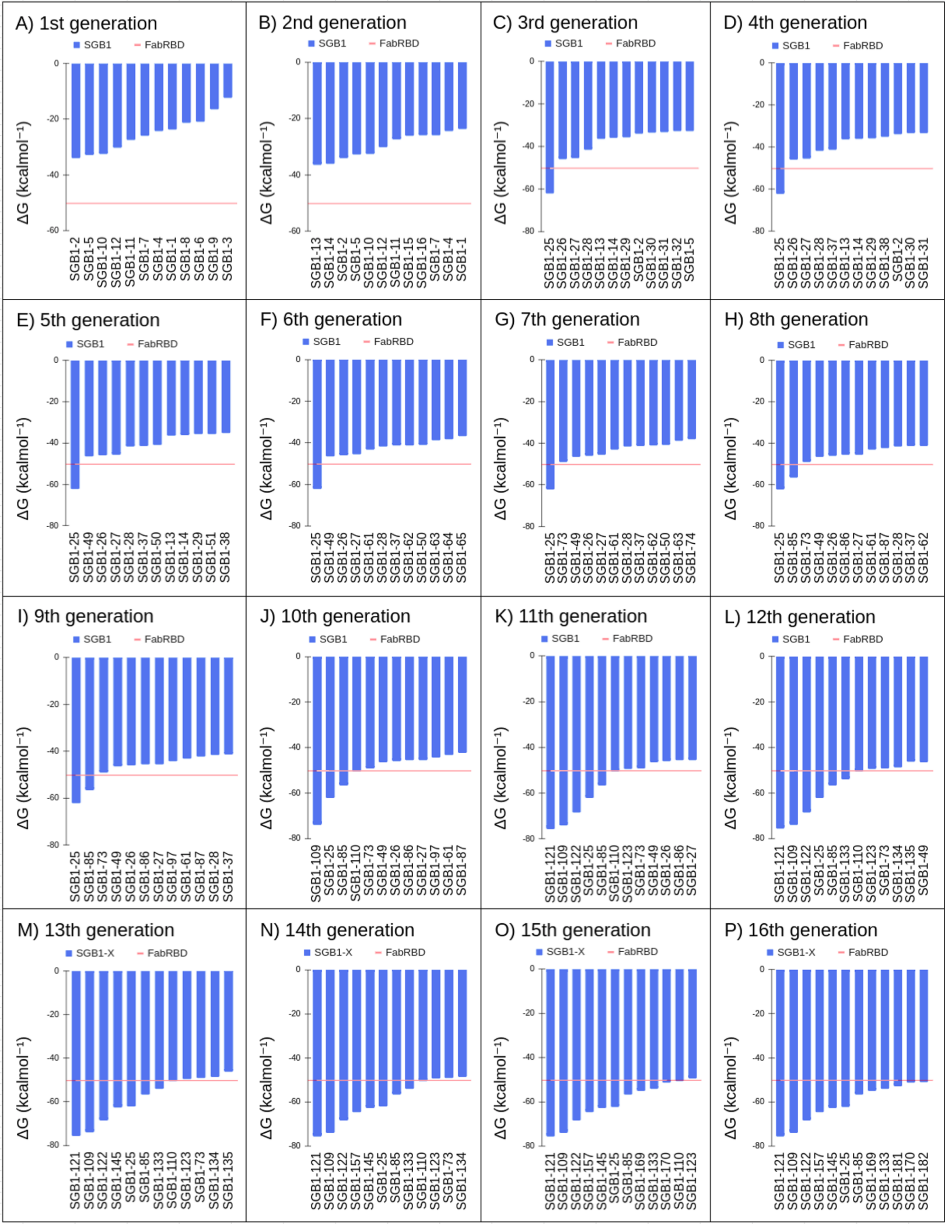
Illustration showing graphs of calculated energies across all GA generations, from (**A**) the first generation to (**P**) the 16th generation.

With the definition of the target of interest, we applied the GA developed by Santo and Feliciano (2021) to optimize the molecular recognition capacity of the GB1 domain. From an initial population constructed with a scoring function, the GA managed to converge the population within just 16 generations. The result can be seen in Figure 3.

In Figure 3A, we can see that all mutants from the initial population have a negative Δ*G*_bind_, indicating some affinity for the antigen. The scoring function achieves the objective of generating an initial population with favorable energy for molecular recognition. However, these energies remain well above the convergence criterion of −50.23 kcal.mol^–1^.

The first mutant with energy below the convergence criterion appears in the third generation (Table S5), and the second mutant only in the eighth generation (Table S15). Due to the much more stringent convergence criterion, convergence occurs much more slowly. however, it is continuous when considering the population average over generations.

From the tenth generation onward, mutants with energies below the convergence criterion begin to emerge more frequently, with the GA converging in the 16th generation. Throughout these 16 generations, the GA favors the formation of the Q4E6R8E10 microdomain, as can be seen in Table S31. It is interesting to note in Table S31 that the original GB1 obtains a negative Δ*G*_bind_, even though it has no known correlation with the RBD domain. However, this result is misleading because, as we have seen in over 200 MDs carried out in the GA, this energy disregards entropic terms, being merely an artifact resulting from the steric compatibility between RBD and GB1.

The mutant SGB1–121, emerging in the 11th generation, obtained an energy of −75.6 kcal.mol^–1^, being the best energy within the final population. As highlighted in Section S3, this result further demonstrates that it is not necessary to wait for the total convergence of the GA to obtain reliable mutants.

When analyzing the convergence criterion using the Δ*G*_bind_ per unit area, as shown in Table S32, we observe that B38 antibody ascends in the ranking, reaching values equivalent to SGB1–25, although still inferior to the mutant SGB1–121. This metric demonstrates greater clarity in comparing the results and can be adopted in future iterations of the GA.

It is important to highlight that the mutant SGB1–121, ranked highest during the GA, obtains better energy than that calculated for mutant peptides from the ACE2 domain, developed by Sitthiyotha and Chunsrivirot (2021),^40^ using the same MM-GBSA tool from AMBER. This result demonstrates the theoretical potential of GB1-based MA, capable of binding to the antigen with better affinity than peptides that are natural ligands of the RBD domain.

#### Energy Decomposition of the SGB1–121 Mutant

**Figure 4:**
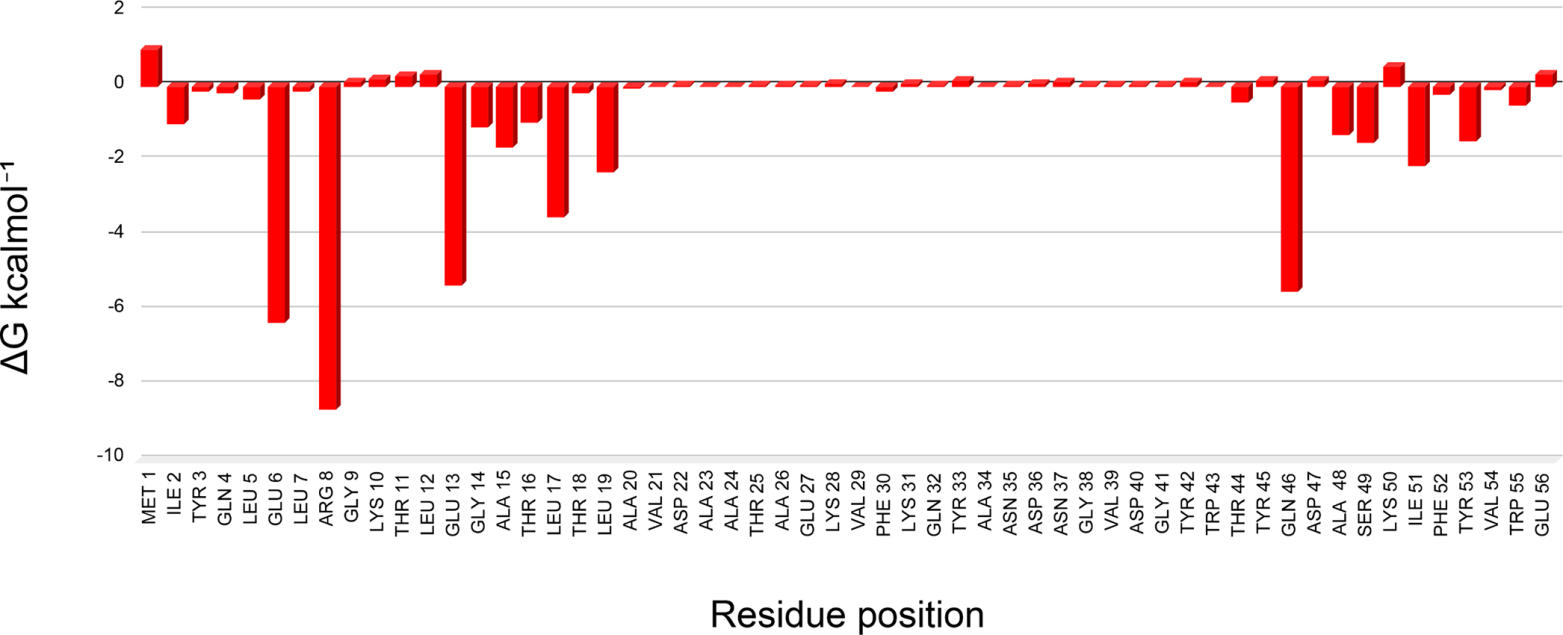
Individual contributions of the residues of the SGB1–121 mutant to the total Δ*G*_bind_.

The Amber energy decomposition tool was applied to the SGB1–121 mutant, and the results can be seen in the graph in Figure 4:

In Figure 4, the energy decomposition analysis of the SGB1–121 mutant reveals several residues crucial for molecular recognition, such as glutamate at positions 6 and 13, arginine at position 8, and glutamine at position 46, all with values below −5 kcal.mol^–1^. Additionally, there is a higher energy contribution from residues near the N-terminal, indicating the possibility of further optimizing the total energy with continued GA iterations.

Due to the highly favorable energetic contribution, we analyzed the structure of Arginine 8 in the complex formed by SGB1–121 and RBD (See Figure S9). Upon examining Arginine in position 8 of SGB1–121, we observed that it is situated within a pocket formed by two α-helices. In this region, its side chain can form hydrogen bonds with residues ASP 71 and GLU 74 of the RBD, which explains the extremely favorable energetic contribution. In addition, one of the nitrogens in its side chain can serve as a connecting bridge between GLU 74 and GLU 13 of the structure of SGB1–121 itself, playing a role that could be performed by a structural water molecule.^41^

This unusual interaction is so surprising that it can only be found by AI algorithms. Due to its ability to indicate the optimization stage of MA by GA, the energy decomposition tool could be used as a convergence criterion in cases where the natural ligands are unknown. Therefore, there are two possibilities to evaluate the quality of optimization within the GA protocol.

### Experimental Validation

#### Antigenic Affinity Assessment

**Figure 5:**
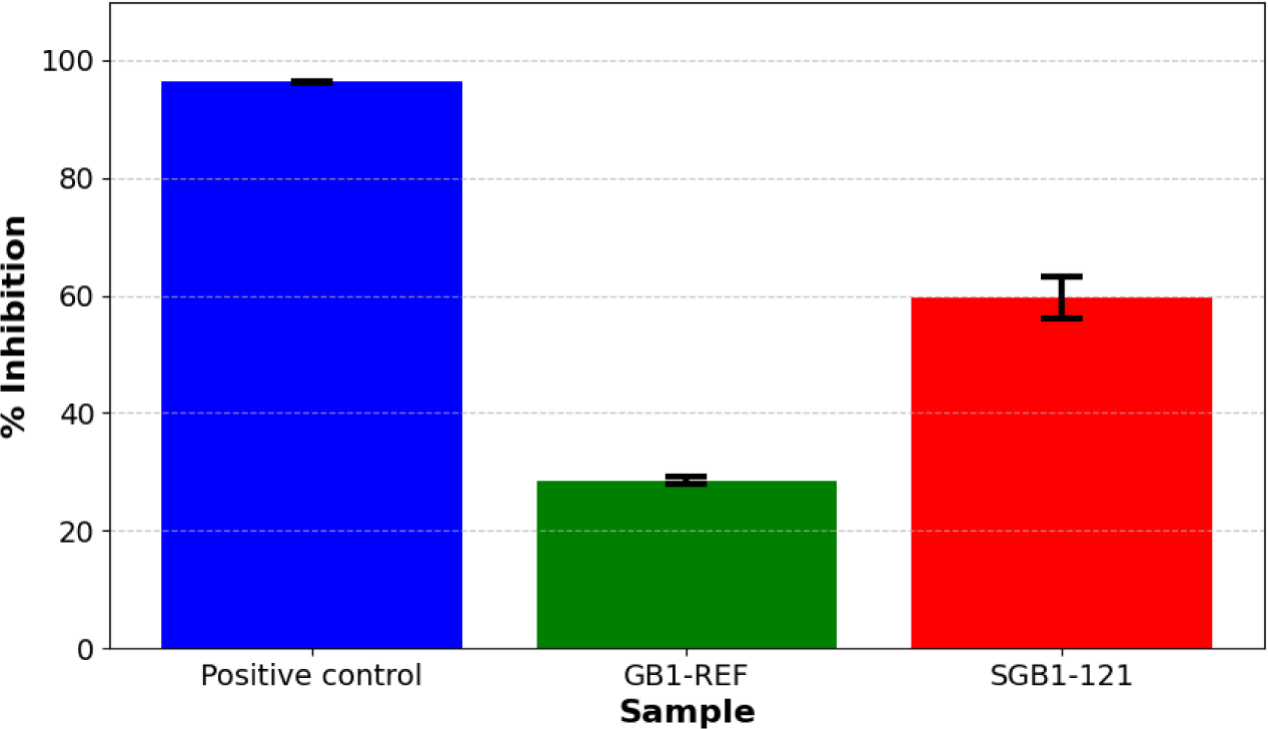
Chart showing the percentage of inhibition of purified extracts containing GB1-REF and SGB1–121. Inhibition values above 30% are considered positive. All samples are normalized using the cPass negative control.

To experimentally confirm the ability of MA to neutralize RBD, we performed the commercially available cPass ELISA kit, purchased from GenScript, the protocol of which is shown in the SI. The cPass was applied with the purified solutions of 55.0*ng*.*μ L*^–1^ mutant SGB1–121_*his-tag*_ and 59.5 _ng_.*μ L*^–1^ of GB1-REF, used for comparison. The test results can be seen in Figure 5:

As shown in Figure 5, the cPass assay data indicate an inhibition percentage of 28.51% ± 0.57% for the GB1-REF and 96% ± 0.08% for the anti-RBD antibody provided in the cPass kit (positive control). The SGB1–121 inhibitor exhibited an inhibition of 59.64% ± 3.61%. For reference, the B38 antibody demonstrated 100% inhibition at concentrations above 2 *ng*.*μ L*^–1^, as described in a previous study by Wu and collaborators (2020).^37^ As detailed in the methodology (See Section S1.8), the cPass test conducted individually is only qualitative, and values above 30% are considered positive for inhibition. All samples are normalized using the cPass negative control. Therefore, the result can be considered positive for the SGB1–121 mutant.

The preliminary results are promising, and at this point, we should emphasize the importance of conducting more tests to increase the reliability of the results, thus allowing for a more robust conclusion. However, the enormous potential for application in MA design is clear, with molecules with therapeutic potential being designed in a matter of weeks, transforming traditional tools for studying biological systems into active agents in the creation of new biotechnological products. All molecular sequences generated by our GA are publicly available and can be found in the SI. We actively encourage the community to reproduce our experiments, fostering transparency and collaboration in advancing research.

### Insights

The MM-GBSA energy decomposition tool can provide information about the most critical residues for molecular recognition, serving as a marker for the level of optimization of the MA within the GA. This information can be very useful in cases where there are no natural ligands to estimate a convergence criterion.

With greater computational power, the protocol can be easily implemented for traditional antibody structures, which are more easily tolerated in organisms. This possibility would allow testing new personalized medicines for selected targets in a matter of months.

The general idea of this study is quite intuitive and easy to understand. In fact, it is just a new approach to using basic protein MD protocols. The main differentiator lies in the modular structure in which the protocols are executed, in the form of a search and optimization algorithm capable of reprogramming the structure and function of GB1.

The methodology used in this study was developed during the execution of the GA and obviously can be improved. One of the most obvious improvements would be the ability to use MD simulations with longer times, or multiple simulations with the same mutant, to better sample conformational possibilities, thus increasing the reliability of the result. The structural and conformational analysis currently performed visually could be automated, allowing for the continuous execution of the GA.

It should be noted that, as with any other computational approach, experimental validation is always necessary to prove biological activity. Preliminary immunogenicity tests of the SGB1–121 mutant indicate a positive result, although caution is necessary due to the need to carry out more experiments.

In this study we sought to work only with highly reliable structures, solved experimentally. In this way, we avoid uncertainties normally related to conventional molecular modeling and docking models. One of the biggest limitations of our technique is related to the MD sampling time. However, as demonstrated by Santo and Feliciano (2021), the most promising microdomains are transmitted to future generations, equivalent to MDs of thousands of ns.

In this context, we opted for a simpler and more streamlined model, focusing mainly on optimizing molecular recognition capacity and improving computational efficiency. The greatest risk in this type of modeling lies in the folding of the MA, where we assume that the template is sufficiently stable (see Section S4). However, since GA produces dozens of potential MAs, there is a chance that some will fold correctly, fulfilling the role for which they were designed.

The GA protocol demonstrates that, when using the MD and MM-GBSA techniques under ideal performance conditions, combined with experimentally reliable structures, it is capable of generating robust results quickly and abundantly. The protocol allows for formidable portability, relying only on software widely used in MD, and is easily auditable at all stages, guaranteeing the transparency necessary for scientific methodology.

A search of the most recent literature shows that docking approaches such as RifDock^24^ and MutDock^25^ also employ the strategy of using rotamers to optimize affinity to the antigen surface. However, in the first approach using RifDock, there is no fixed backbone for MA in principle, and each ligand molecule can be completely different. In the MutDock approach, although very similar to our initial population construction, there was still experimental validation. The most significant innovation in the methodology developed in this work is, without a doubt, the use of GA.

The research demonstrates the transformative potential of GA in the field of biotechnology, specifically in the design of MA. The integration of GA allows not only to accelerate the development process, but also to increase the precision and efficiency of the resulting therapeutic agents.

## Conclusion

In this study, we present an approach that demonstrates that MA modeling can be performed using GA-based AI. Such algorithms make use of a combination of software widely used in molecular simulation. These software programs, when structured in the form of a GA, can redefine the role of the GB1 domain. Such reconfiguration, achieved through the reprogramming of its structure and function, holds significant potential to guide experimental research for new bioactive molecules. The execution of the GA is computationally feasible due to the choice of a structure for MA and antigenic epitopes with extremely small dimensions, yet capable of maintaining structural stability.

The computational results indicate that, using the methodology in this study, it is possible to develop structural motifs capable of neutralizing antigens. These structural motifs can be designed based on the MA structure itself, in an original and innovative way, without the need for structural prediction software, molecular docking, or preexisting databases. The generated mutants reconcile the high stability of GB1 with the optimized properties of MA. Therefore, we can state that the objective of developing a stable peptide base for conversion into MA has been achieved.

The experimental antigenic affinity tests show a positive outcome, confirming that the SGB1–121 antibody can bind to the RBD antigen for which it was designed. Although these results are not yet definitive, requiring further experiments, the viability of the methodology used in this study for developing MA is evident. All procedures can be completed in significantly shorter periods than conventional searches through extensive databases of bioactive molecules. Consequently, we can assert that the experimental validation phase yielded satisfactory results, demonstrating that the objectives of this study have been achieved.

## Data Availability

The software and data are available on GitHub https://github.com/gallactuz/Data-and-Software-Availability.

## Supporting Information Available

Additional experimental details, materials, and methods, including photographs of the experimental setup, tables containing results from MD simulations, and figures with additional analyses (PDF)
