# Supplementary material for: Design of Mimetic Antibodies Targeting the SARS-CoV-2 Spike Glycoprotein Based on the GB1 Domain: A Molecular Simulation and Experimental Study
Source: Biochemistry. 2025 Mar 17;64(7):1541–9. doi: 10.1021/acs.biochem.4c00671 (PMC11966750; doi:10.1021/acs.biochem.4c00671)
Supplement: Supplementary file 1 — bi4c00671_si_001.pdf [file bi4c00671_si_001.pdf]

Supporting Information
Design of Mimetic Antibodies Targeting the SARS-CoV-2 Spike Glycoprotein Based on the GB1 Domain: A Molecular Simulation and Experimental Study

...,[†] Anderson A. E. Santo,[†] Aline Reis,[‡] Anderson A. Pinheiro,[‡] Paulo I. da Costa,[*,‡] and Gustavo T. Feliciano[*,†]

†*Institute of Chemistry, São Paulo State University, Araraquara, SP, 14800-900, Brazil*

‡*School of Pharmaceutical Sciences, São Paulo State University, Araraquara, SP, 14801-360, Brazil*

E-mail: paulo-inacio.costa@unesp.br; gustavo.troiano@unesp.br

Contents

S1 Experimental protocols **S3**

S1.1 Cells, proteins, genes and vectors . . . S3

S1.2 Cell preparation and transformation . . . S3

S1.3 Plasmid DNA extraction (Miniprep) . . . S5

S1.4 Gene amplification . . . S5

S1.5 Growth curve . . . S8

S1.6 Protein expression and purification . . . S9

S1.7 Immunodot . . . S11

S1.8 Competitive ELISA . . . S12

S2 Energies of the genetic algorithm for anti-RBD SGB1 **S13**

S3 $\Delta G_{\mathbf{bind}}$ per unit of contact area analysis **S29**

S4 GA Standard deviation **S31**

S5 Structural analysis **S33**

References **S34**

S1 Experimental protocols

S1.1 Cells, proteins, genes and vectors

The *E. coli* DH5α[1] lineage (vector propagation) and *E. coli* BL21 (DE3)[2] Rosetta lineage (protein expression) were acquired from Thermo Fisher Scientific and were maintained in stock in an ultrafreezer at -80°C. The synthetic genes comprising GB1-REF and GB1-121 were purchased from GenOne Brasil (Rio de Janeiro, RJ), cloned into the pET-28a(+) expression vector (pET-28a/GB1-REF and pET-28a/GB1-121) between the NdeI and XhoI sites for the N-terminal and C-terminal ends, respectively. The type 2 human angitensin-converting enzyme (hACE2) was purchased from Sigma (Saint Louis, USA).

S1.2 Cell preparation and transformation

The transformation of bacterial cells with the pET-28a/GB1-REF or pET-28a/GB1-121 vectors was achieved using the calcium chloride method with minor modifications.[3] Briefly, 5 mL of Luria-Bertani (LB) medium was inoculated with *E. coli* cells from the laboratory's collection and incubated overnight at 37 °C and 240 rpm. On the next day, 5 mL of fresh LB broth was inoculated with 50 μL of the overnight culture and allowed to grow for exactly 2 hours to reach an optical density (OD) of 0.4. Then, cells were centrifuged at 4 ºC and 4500 rpm for 10 minutes, the supernatant was discarded, the pellet was washed twice with cold 0.1 M calcium chloride solution, resuspended in the same solution and kept on ice for 30 minutes. After this incubation, the suspension was centrifuged again, the supernatant discarded, and the cells were gently resuspended in 300 µL of 0.1 M $CaCl_2$.

For transformation, approximately 50 ng of the recombinant plasmid (pET-28a/GB1-REF or pET-28a/GB1-121) was added to aliquots of competent cells. The reaction was kept on ice for 25 minutes and then subjected to a thermal shock for 90 seconds in a 42 °C water bath, and immediately returned to the ice bath for an additional 5 minutes. After the thermal shock, 1 mL of fresh LB medium was added to each tube, and the bacteria were

then incubated in a shaker at 37 °C for about 1 hour with agitation at 240 rpm for recovery. After recovery, selection was performed by plating on solid LB-Agar medium with 25 mg/mL of Kanamycin (KAN). The plates were incubated at 37°C for about a day or until colonies developed.

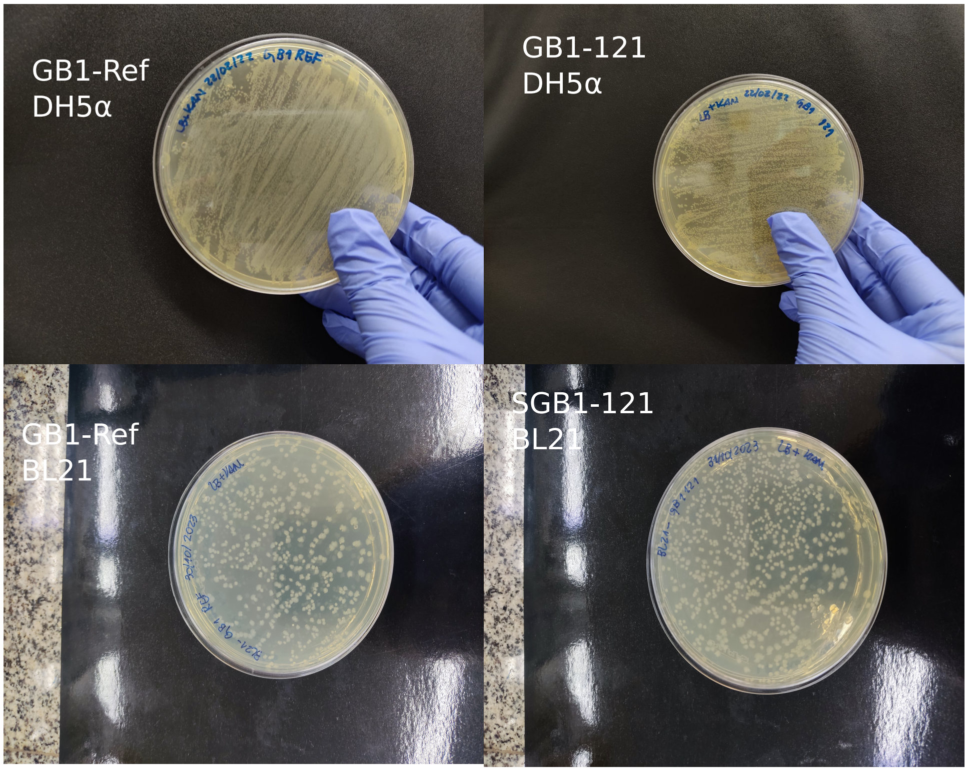

Figure S1: Transformant selection on LB-Agar plates with 25 mg/mL KAN.

S1.3 Plasmid DNA extraction (Miniprep)

The miniprep was performed using the Concert™ Rapid Plasmid Maxiprep System kit (Life Technologies) following the manufacturer's instructions. Briefly, a colony of *E. coli* DH5α carrying the recombinant vector pET-28a/GB1-REF or pET-28a/GB1-121 was grown in 5 mL of LB medium with 25 mg/mL KAN overnight at 37 °C and 240 rpm. From this culture, 3 mL were centrifuged at and 12000 xg for 5 minutes, the supernatant was discarded, and the cells were resuspended in 210 μL of buffer G1 with RNase A. Next, 210 μL of buffer G2 was added, the contents were mixed by inversion, and the tube was kept at room temperature for 5 minutes. Then, 280 μL of solution G3 was added, the tube was mixed by inversion, and then centrifuged for 10 minutes at 12000 xg. The supernatant was transferred to the binding column assembly with collection tube and centrifuged at 12000 xg for 1 minute. The contents of the collection tube were discarded, and the column was washed with 750 μL of buffer G4. After washing, the column was attached to a DNAse and RNAse-free 1.5 mL microcentrifuge tube, and the plasmid DNA was eluted by adding 50 μL of TE buffer. The extracted plasmid DNA was quantified using a NanoVue Plus Spectrophotometer (Biochrom) and stored in a freezer at -20 °C.

S1.4 Gene amplification

To confirm the identity of the extracted plasmids, gene amplification was performed through conventional PCR and the amplified products were visualized by agarose gel electrophoresis. For this purpose, 1 μL of template DNA (pET-28a/GB1-REF or pET-28a/GB1-121) and 0.2 μL of Taq DNA polymerase were added to 20 μL of the Master Mix (Nuclease Free Water; commercial PCR buffer; 1.5 mM $MgCl_2$; 0.5 μM Prime Forward; 0.5 μM Primer Reverse; 0.2 mM dNTPs) and subjected to thermal cycling in a Geneamp 9700 PCR Thermal Cycler (Applied Biosystems) according to Table S0.

Table S0: PCR Cycling Parameters.

Cycles	Phase	Temperature	Time
1x	Stabilization	94 ºC	3-5 min
30x	Denaturation	94 ºC	45 sec
30x	Annealing	55 ºC	30 sec
30x	Extension	72 ºC	1 min 30 sec
1x	Final Extension	72 ºC	5 min
1x	Maintenance	4 ºC	∞

Once the PCR was completed, a 1.5% agarose gel was prepared by diluting 1.5 g of agarose in 100 mL of TBE buffer (0.1 M Tris base; 0.09 M Anhydrous Boric Acid; 1 mM Sodium EDTA). The still liquid gel was poured into the mold, and the comb was inserted. After gel polymerization, the comb was removed, and the tank was filled with 1X TBE. Subsequently, the molecular ruler and the sample were applied to the wells. A potential difference of 100 V was applied for electrophoresis until the migration front approached the middle of the gel. At the end of the run, the gel was placed in a L-PIX Transilluminator (Loccus), and an image was captured for analysis.

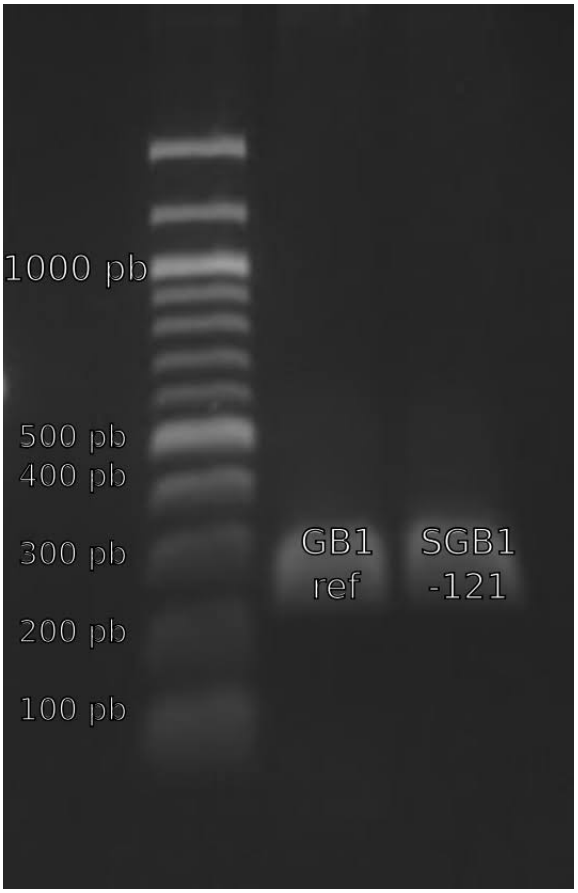

Figure S2: Electrophoretic analysis on 1.5% agarose gel of the amplification products.

In Figure S2, we can see the molecular sizes of the amplified fragments corresponding to the GB1 reference and the SGB1-121 mutant. It is expected that both GB1-REF and SGB1-121 fall within the size range of 186 bp. However, the primers used (T7 Promoter (Forward) and T7 Terminator (Reverse)) flank a region of $\tilde{3}00$ bp at the plasmid cloning site. This result indicates that the library contains the genes of interest, with bands corresponding to their molecular weights, indicating that the bacterial transformation was successful.

S1.5 Growth curve

Before proceeding with the production of recombinant proteins, it was necessary to evaluate the growth profile of the *E. coli* BL21 (DE3) Rosetta strain carrying the pET-28a/GB1-REF and pET-28a/GB1-121 vectors. A pre-inoculum was prepared using each of the cultures, which were grown overnight in 5 mL of liquid LB medium with 25 mg/mL Kanamycin. An aliquot of 1 mL of those cultures were then inoculated into 100 mL of liquid LB medium with KAN. Both cultures were incubated in a shaker at 240 rpm and 37 °C, and the optical density at 600 nm was monitored every 10 minutes using a Biowave II UV-Vis Spectrophotometer (WPA).

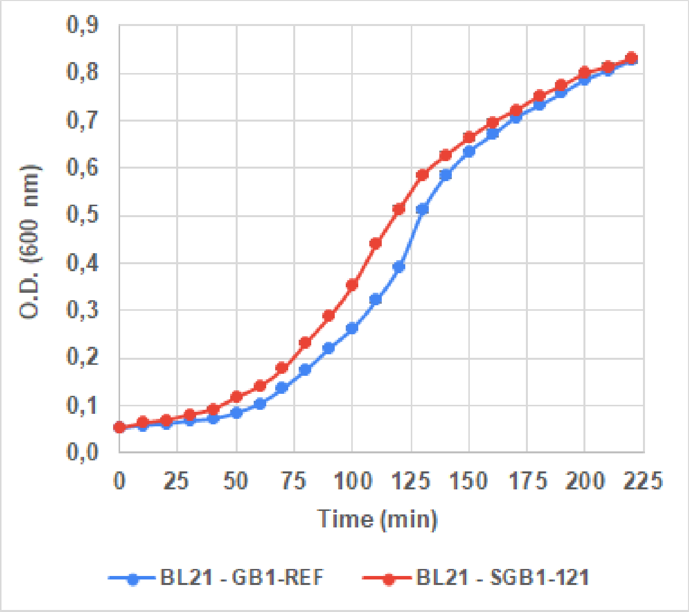

Figure S3: Growth profile comparison between *E. coli* BL21 (DE3) carrying pET-28a/GB1-REF or pET-28a/GB1-121 in selective media with 25 mg/mL KAN.

S1.6 Protein expression and purification

For protein expression, a pre-inoculum was prepared using *E. coli* BL21 (DE3) strain carrying pET-28a/GB1-REF and pET-28a/GB1-121 vectors, which were grown overnight in 5 mL of LB broth with 25 mg/mL Kanamycin at 37 °C and 240 rpm. On the next day, 150 mL of fresh LB medium supplemented with KAN was inoculated with 3 mL from the overnight cultures, and were grown to an O.D. of 0.4 to 0.6. Expression was then induced with the addition of IPTG (isopropyl β-D-1-thiogalactopyranoside) to a final concentration of 1 mM, and cell cultures remain incubated for 4 hours at 28 °C with shaking at 240 rpm.

The cellular biomass was harvested by centrifugation step at 1600 xg for 10 minutes at 4 °C. The supernatant was discarded, and the cell mass was washed with washing buffer (Tris-HCl 100 mM and EDTA 10 mM). For obtaining the protein extract, the pellet was resuspended in 3 mL of lysis buffer (NaCl 0,01 M; NaH_2PO_4 0,05 M; PMSF 0,001 M; Tris-HCl 0,01 M, EDTA 0,001 M and DTT 5 mM) and subjected to cell disruption by sonication for a period of 5 minutes, with 30-second intervals, while keeping the tubes in ice. Subsequently, the lysate was centrifuged at 2200 xg for 10 minutes at 4 °C, allowing the separation of the supernatant, which was reserved, and the pellet, which was discarded. The protein extracts were stored in a freezer at -20 °C.

Protein purification was achieved using a HisTrap™ chromatographic column with Sepharose™ Fast Flow-Ni^{2+}, which has an affinity for the His6x tag fused to the C-terminal end of both GB1-REF and GB1-121. The column was washed with 15 mL of ultrapure water to remove the storage solution (20% ethanol). Next, it was equilibrated by introducing 25 mL of binding solution (NaH_2PO_4 20 mM; NaCl 500 mM; and imidazole 20 mM) to prepare the chromatographic bed. The supernatant fraction from the cell lysates, supplemented with 20 mM imidazole, were filtered through a 0.22 μm membrane and then applied to the column. To remove unbound proteins, a wash with 50 mL of binding solution was performed. Elution of the bound proteins occurred in a single step by injecting 50 mL of elution solution (NaH_2PO_4 20 mM; NaCl 500 mM; and imidazole 500 mM).

For the construction of the chromatographic profile, samples were collected every 5 mL eluted from the column. The protein concentration was determined using the Micro BCA^{TM} Protein Assay Kit (Thermo Scientific), following the manufacturer's instructions. Briefly, 100 μL of each sample or protein standard were added to wells of a microplate, and 100 μL of the working reagent was also added. The plate was shaken for 30 seconds, sealed, and then incubated at 37 °C for a period of 2 hours. After the incubation was completed, absorbance was measured at 562 nm using the GO accuSkan microplate reader (Fisher Scientific).

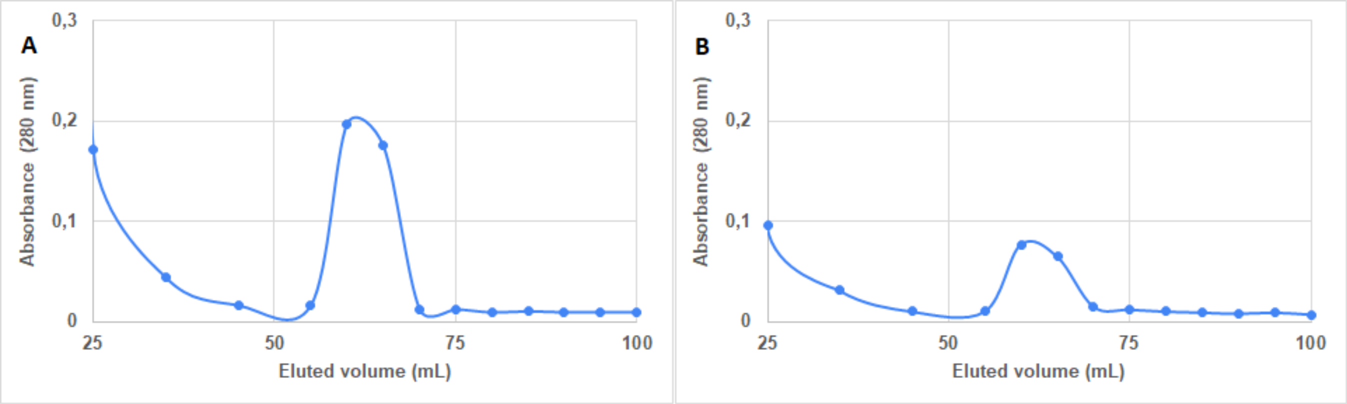

Figure S4: Representative purification chromatograms of GB1-REF (A) and SGB1-121 (B).

S1.7 Immunodot

To confirm protein expression and evaluate their reactivity, an immunodot was performed. Briefly, 3 μL of each sample were applied onto nitrocellulose membranes, which were subsequently air-dried at room temperature and transferred to 2 mL tubes. For the blocking procedure, the membranes were treated with a blocking solution (PBS buffer with 1.5% Albumin) for a period of 2 hours. After blocking, the membranes were incubated with a solution containing the anti-6xHisTag monoclonal antibody conjugated with HRP (3D5) (Thermo Fisher, Catalog: R931-25) at a dilution of 1:3000, overnight at room temperature. On the following day, the membranes were then washed three times with PBS-Tween (0.005%) and subjected to visualization with a DAB solution (0.06%) and hydrogen peroxide (0.03%) in PBS. Once the bands appeared, the membranes were washed with purified water to stop the visualization reaction, and excess water was removed with filter paper. The membranes were then photographed.

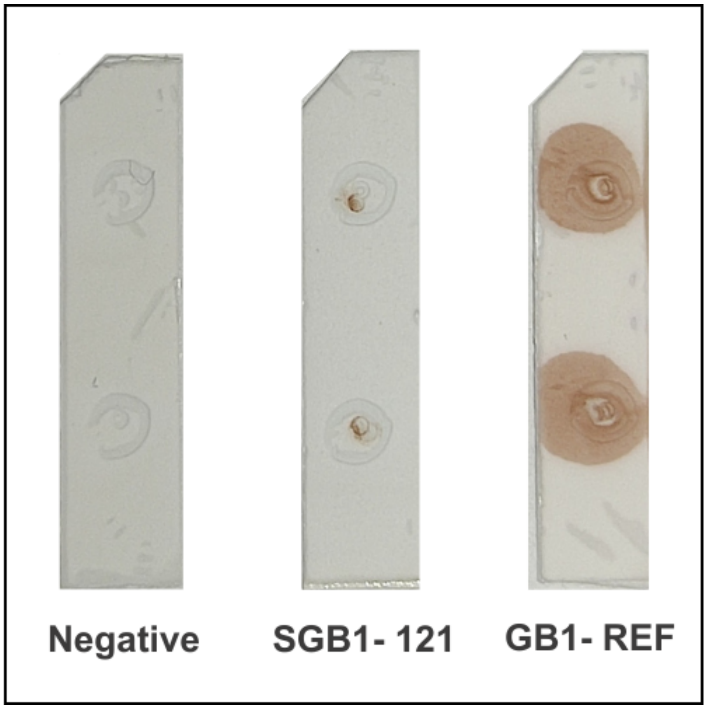

Figure S5: Immunodot for expression confirmation of GB1-REF and SGB1-121.

S1.8 Competitive ELISA

In this ELISA assay, the wells are coated with the ACE2 receptor, and the RBD is conjugated to horseradish peroxidase (HRP). Upon addition of the RBD-HRP complex and the test antibodies, if the antibodies fail to neutralize the RBD, it will bind to the ACE2 immobilized on the plate. The HRP enzyme linked to the RBD will then catalyze a substrate reaction, producing a color change. Conversely, if the antibodies effectively neutralize the RBD, it will be unable to bind to the ACE2 receptor, resulting in reduced or no color development. The intensity of the color directly correlates with the extent of RBD-ACE2 binding and thus reflects the neutralizing capacity of the antibodies. To confirm the inhibitory activity of the designed GB1-121, the cPass^{TM} SARS-CoV-2 Neutralization Antibody Detection Kit was used following the manufacturer's instructions with minor modifications. First, 60 μL of samples and controls were pre-incubated with 60 μL of HRP-RBD solution for 2 hours at 37 °C using a round bottle microplate without binding properties. Then, 100 μL of the mixture was added to the capture plate pre-coated with hACE2 protein and incubated for 1 hour at 37 °C. Unbound HRP-RBD is captured on the plate while neutralized antibody/HRP-RBD complexes remain in suspension and are removed during washing. The plates were washed 4x with washing solution, and 100 μL TMB solution was added and the plates incubated for 15 minutes at room temperature. Then, 50 μL of stop solution (1 M sulfuric acid) was added to extinguish the reaction, and the plates were read at 450 nm using the GO accuSkan microplate reader (Fisher Scientific). The inhibitory activity can be calculated following the equation:

% Inhibition = (1 - (Sample ABS Value / Negative Control ABS Value)) x 100

A $\geq$ 30% inhibition indicates SARS-CoV-2 neutralizing activity (positive result), while $<$ 30% inhibition indicates no SARS-CoV-2 neutralizing activity (negative result).

S2 Energies of the genetic algorithm for anti-RBD SGB1

Table S1: First generation of anti-RBD SGB1. All values of ΔG are kilocalories per mole.

Code \ Resid	2	4	6	8	13	15	17	19	42	44	46	49	51	53	55	ΔG
FabRBD	X	X	X	X	X	X	X	X	X	X	X	X	X	X	X	-50.2
SGB1-2	E	I	Y	T	M	L	Y	I	L	I	W	I	A	R	M	-34.0
SGB1-5	I	M	E	N	K	Y	H	E	N	L	R	K	S	E	L	-32.8
SGB1-10	N	K	T	A	L	W	N	S	T	H	N	Q	L	V	I	-32.6
SGB1-12	S	N	S	H	T	I	A	G	P	N	P	P	R	S	S	-30.3
SGB1-11	T	T	V	Y	I	M	D	T	W	S	A	H	P	T	H	-27.4
SGB1-7	H	R	L	E	V	D	V	Q	R	Q	M	N	Y	H	N	-26.0
SGB1-4	W	H	R	I	R	T	K	W	Y	K	Q	S	I	Y	W	-24.4
SGB1-1	Y	L	W	M	E	A	L	L	Q	W	H	A	G	W	Y	-23.8
SGB1-8	Q	Q	Q	R	H	E	T	M	K	D	I	D	N	Q	Q	-21.3
SGB1-6	R	E	I	Q	W	N	W	R	A	T	E	M	T	I	T	-21.0
SGB1-9	M	S	N	L	P	H	E	V	S	V	L	G	E	M	R	-16.4
SGB1-3	K	V	H	V	A	V	R	Y	V	Y	Y	L	V	K	K	-12.4

Table S2: First generation children. All values of ΔG are kilocalories per mole.

Code \ Resid	2	4	6	8	13	15	17	19	42	44	46	49	51	53	55	ΔG
SGB1-13	Y	L	W	M	E	A	L	L	Y	K	Q	S	I	Y	W	-36.5
SGB1-14	Q	Q	Q	R	H	E	T	M	A	T	E	M	T	I	T	-36.1
SGB1-15	E	I	Y	T	M	L	Y	I	N	L	R	K	S	E	L	-26.2
SGB1-16	S	N	S	H	T	I	A	G	T	H	N	Q	L	V	I	-26.0
SGB1-17	I	M	E	N	K	Y	H	E	L	I	W	I	A	R	M	-24.8
SGB1-18	T	T	V	Y	I	M	D	T	R	Q	M	N	Y	H	N	-23.8
SGB1-19	H	R	L	E	V	D	V	Q	W	S	A	H	P	T	H	-21.4
SGB1-20	K	V	H	V	A	V	R	Y	S	V	L	G	E	M	R	-14.5
SGB1-21	M	S	N	L	P	H	E	V	V	Y	Y	L	V	K	K	-8.2
SGB1-22	R	E	I	Q	W	N	W	R	K	D	I	D	N	Q	Q	-7.9
SGB1-23	W	H	R	I	R	T	K	W	Q	W	H	A	G	W	Y	-0.7
SGB1-24UNFOLD	N	K	T	A	L	W	N	S	P	N	P	P	R	S	S	-41.2

Table S3: Second generation of anti-RBD SGB1. All values of ΔG are kilocalories per mole.

Code \ Resid	2	4	6	8	13	15	17	19	42	44	46	49	51	53	55	ΔG
FabRBD	X	X	X	X	X	X	X	X	X	X	X	X	X	X	X	-50.2
SGB1-13	Y	L	W	M	E	A	L	L	Y	K	Q	S	I	Y	W	-36.5
SGB1-14	Q	Q	Q	R	H	E	T	M	A	T	E	M	T	I	T	-36.1
SGB1-2	E	I	Y	T	M	L	Y	I	L	I	W	I	A	R	M	-34.0
SGB1-5	I	M	E	N	K	Y	H	E	N	L	R	K	S	E	L	-32.8
SGB1-10	N	K	T	A	L	W	N	S	T	H	N	Q	L	V	I	-32.6
SGB1-12	S	N	S	H	T	I	A	G	P	N	P	P	R	S	S	-30.3
SGB1-11	T	T	V	Y	I	M	D	T	W	S	A	H	P	T	H	-27.4
SGB1-15	E	I	Y	T	M	L	Y	I	N	L	R	K	S	E	L	-26.2
SGB1-16	S	N	S	H	T	I	A	G	T	H	N	Q	L	V	I	-26.0
SGB1-7	H	R	L	E	V	D	V	Q	R	Q	M	N	Y	H	N	-26.0
SGB1-4	I	M	E	N	K	Y	H	E	L	I	W	I	A	R	M	-24.8
SGB1-1	W	H	R	I	R	T	K	W	Y	K	Q	S	I	Y	W	-24.4

Table S4: Second generation children. All values of ΔG are kilocalories per mole.

Code \ Resid	2	4	6	8	13	15	17	19	42	44	46	49	51	53	55	ΔG
SGB1-25	S	N	S	H	L	W	N	S	P	N	P	Q	L	V	I	-62.2
SGB1-26	I	M	E	N	M	L	Y	I	N	L	R	I	A	R	M	-46.0
SGB1-27	Y	L	W	M	E	A	L	L	A	T	E	M	T	I	T	-45.6
SGB1-28	E	I	Y	T	K	Y	H	E	L	I	W	K	S	E	L	-41.7
SGB1-29	I	M	E	N	K	Y	H	E	Y	K	Q	S	I	Y	W	-35.8
SGB1-30	Q	Q	Q	R	H	E	T	M	Y	K	Q	S	I	Y	W	-33.5
SGB1-31	S	N	S	H	T	I	A	G	R	Q	M	N	Y	H	N	-33.4
SGB1-32	N	K	T	A	T	I	A	G	T	H	N	P	R	S	S	-32.8
SGB1-33	E	I	Y	T	M	L	Y	I	W	S	A	H	P	T	H	-31.6
SGB1-34	W	H	R	I	R	T	K	W	L	I	W	I	A	R	M	-27.3
SGB1-35	T	T	V	Y	I	M	D	T	N	L	R	K	S	E	L	-19.9
SGB1-36	H	R	L	E	V	D	V	Q	T	H	N	Q	L	V	I	-16.9

Table S5: Third generation of anti-RBD SGB1. All values of ΔG are kilocalories per mole.

Code \ Resid	2	4	6	8	13	15	17	19	42	44	46	49	51	53	55	ΔG
SGB1-25	S	N	S	H	L	W	N	S	P	N	P	Q	L	V	I	-62.2
FabRBD	X	X	X	X	X	X	X	X	X	X	X	X	X	X	X	-50.2
SGB1-26	I	M	E	N	M	L	Y	I	N	L	R	I	A	R	M	-46.0
SGB1-27	Y	L	W	M	E	A	L	L	A	T	E	M	T	I	T	-45.6
SGB1-28	E	I	Y	T	K	Y	H	E	L	I	W	K	S	E	L	-41.7
SGB1-13	Y	L	W	M	E	A	L	L	Y	K	Q	S	I	Y	W	-36.5
SGB1-14	Q	Q	Q	R	H	E	T	M	A	T	E	M	T	I	T	-36.1
SGB1-29	I	M	E	N	K	Y	H	E	Y	K	Q	S	I	Y	W	-35.8
SGB1-2	E	I	Y	T	M	L	Y	I	L	I	W	I	A	R	M	-34.0
SGB1-30	Q	Q	Q	R	H	E	T	M	Y	K	Q	S	I	Y	W	-33.5
SGB1-31	S	N	S	H	T	I	A	G	R	Q	M	N	Y	H	N	-33.4
SGB1-32	N	K	T	A	T	I	A	G	T	H	N	P	R	S	S	-32.8
SGB1-5	I	M	E	N	K	Y	H	E	N	L	R	K	S	E	L	-32.8

Table S6: Third generation children. All values of ΔG are kilocalories per mole.

Code \ Resid	2	4	6	8	13	15	17	19	42	44	46	49	51	53	55	ΔG
SGB1-37	Q	Q	Q	R	H	E	T	M	R	Q	M	N	Y	H	N	-41.3
SGB1-38	E	I	Y	T	K	Y	H	E	A	T	E	M	T	I	T	-35.1
SGB1-39	Y	L	W	M	E	A	L	L	L	I	W	K	S	E	L	-32.6
SGB1-40	I	M	E	N	K	Y	H	E	T	H	N	P	R	S	S	-30.4
SGB1-41	E	I	Y	T	K	Y	H	E	L	I	W	S	I	Y	W	-25.2
SGB1-42	Y	L	W	M	H	E	T	M	Y	K	Q	M	T	I	T	-22.6
SGB1-43	S	N	S	H	L	W	N	S	N	L	R	I	A	R	M	-21.1
SGB1-44	Q	Q	Q	R	E	A	L	L	A	T	E	S	I	Y	W	-19.1
SGB1-45	N	K	T	A	T	I	A	G	N	L	R	K	S	E	L	-18.1
SGB1-46	I	M	E	N	M	L	Y	I	Y	K	Q	I	A	R	M	-17.6
SGB1-47	I	M	E	N	M	L	Y	I	P	N	P	Q	L	V	I	-17.3
SGB1-48	S	N	S	H	T	I	A	G	Y	K	Q	S	I	Y	W	-17.2

Table S7: Fourth generation of anti-RBD SGB1. All values of ΔG are kilocalories per mole.

Code \ Resid	2	4	6	8	13	15	17	19	42	44	46	49	51	53	55	ΔG
SGB1-25	S	N	S	H	L	W	N	S	P	N	P	Q	L	V	I	-62.2
FabRBD	X	X	X	X	X	X	X	X	X	X	X	X	X	X	X	-50.2
SGB1-26	I	M	E	N	M	L	Y	I	N	L	R	I	A	R	M	-46.0
SGB1-27	Y	L	W	M	E	A	L	L	A	T	E	M	T	I	T	-45.6
SGB1-28	E	I	Y	T	K	Y	H	E	L	I	W	K	S	E	L	-41.7
SGB1-37	Q	Q	Q	R	H	E	T	M	R	Q	M	N	Y	H	N	-41.3
SGB1-13	Y	L	W	M	E	A	L	L	Y	K	Q	S	I	Y	W	-36.5
SGB1-14	Q	Q	Q	R	H	E	T	M	A	T	E	M	T	I	T	-36.1
SGB1-29	I	M	E	N	K	Y	H	E	Y	K	Q	S	I	Y	W	-35.8
SGB1-38	E	I	Y	T	K	Y	H	E	A	T	E	M	T	I	T	-35.1
SGB1-2	E	I	Y	T	M	L	Y	I	L	I	W	I	A	R	M	-34.0
SGB1-30	Q	Q	Q	R	H	E	T	M	Y	K	Q	S	I	Y	W	-33.5
SGB1-31	S	N	S	H	T	I	A	G	R	Q	M	N	Y	H	N	-33.4

Table S8: Fourth generation children. All values of ΔG are kilocalories per mole.

Code \ Resid	2	4	6	8	13	15	17	19	42	44	46	49	51	53	55	ΔG
SGB1-49	Q	Q	Q	R	E	A	L	L	R	Q	M	S	I	Y	W	-46.5
SGB1-50	Y	L	W	M	H	E	T	M	Y	K	Q	N	Y	H	N	-41.0
SGB1-51	I	M	E	N	H	E	T	M	Y	K	Q	M	T	I	T	-35.7
SGB1-52	Q	Q	Q	R	T	I	A	G	Y	K	Q	N	Y	H	N	-34.0
SGB1-53	S	N	S	H	M	L	Y	I	P	N	P	I	A	R	M	-28.7
SGB1-54	Y	L	W	M	K	Y	H	E	A	T	E	K	S	E	L	-26.3
SGB1-55	I	M	E	N	L	W	N	S	N	L	R	Q	L	V	I	-22.4
SGB1-56	Q	Q	Q	R	K	Y	H	E	A	T	E	S	I	Y	W	-19.2
SGB1-57	S	N	S	H	H	E	T	M	R	Q	M	S	I	Y	W	-22.3
SGB1-58	E	I	Y	T	E	A	L	L	L	I	W	M	T	I	T	-17.9
SGB1-59	E	I	Y	T	K	Y	H	E	L	I	W	I	A	R	M	-3.0
SGB1-60	E	I	Y	T	M	L	Y	I	A	T	E	M	T	I	T	-0.4

Table S9: Fifth generation of anti-RBD SGB1. All values of ΔG are kilocalories per mole.

Code \ Resid	2	4	6	8	13	15	17	19	42	44	46	49	51	53	55	ΔG
SGB1-25	S	N	S	H	L	W	N	S	P	N	P	Q	L	V	I	-62.2
FabRBD	X	X	X	X	X	X	X	X	X	X	X	X	X	X	X	-50.2
SGB1-49	Q	Q	Q	R	E	A	L	L	R	Q	M	S	I	Y	W	-46.5
SGB1-26	I	M	E	N	M	L	Y	I	N	L	R	I	A	R	M	-46.0
SGB1-27	Y	L	W	M	E	A	L	L	A	T	E	M	T	I	T	-45.6
SGB1-28	E	I	Y	T	K	Y	H	E	L	I	W	K	S	E	L	-41.7
SGB1-37	Q	Q	Q	R	H	E	T	M	R	Q	M	N	Y	H	N	-41.3
SGB1-50	Y	L	W	M	H	E	T	M	Y	K	Q	N	Y	H	N	-41.0
SGB1-13	Y	L	W	M	E	A	L	L	Y	K	Q	S	I	Y	W	-36.5
SGB1-14	Q	Q	Q	R	H	E	T	M	A	T	E	M	T	I	T	-36.1
SGB1-29	I	M	E	N	K	Y	H	E	Y	K	Q	S	I	Y	W	-35.8
SGB1-51	I	M	E	N	H	E	T	M	Y	K	Q	M	T	I	T	-35.7
SGB1-38	E	I	Y	T	K	Y	H	E	A	T	E	M	T	I	T	-35.1

Table S10: Fifth generation children. All values of ΔG are kilocalories per mole.

Code \ Resid	2	4	6	8	13	15	17	19	42	44	46	49	51	53	55	ΔG
SGB1-61	I	Q	E	R	H	Y	T	E	Y	T	Q	S	T	Y	T	-43.2
SGB1-62	Y	L	W	M	H	E	T	M	Y	K	Q	S	I	Y	W	-41.2
SGB1-63	Y	L	W	M	E	A	L	L	N	L	R	I	A	R	M	-38.8
SGB1-64	Y	L	W	M	E	A	L	L	Y	K	Q	N	Y	H	N	-38.2
SGB1-65	Q	Q	Q	R	E	A	L	L	P	N	P	Q	L	V	I	-36.7
SGB1-66	E	I	Y	T	K	Y	H	E	R	Q	M	N	Y	H	N	-34.7
SGB1-67	Q	Q	Q	R	H	E	T	M	L	I	W	K	S	E	L	-28.0
SGB1-68	I	M	E	N	H	E	T	M	A	T	E	M	T	I	T	-27.3
SGB1-69	I	M	E	N	M	L	Y	I	A	T	E	M	T	I	T	-24.0
SGB1-70	S	N	S	H	L	W	N	S	R	Q	M	S	I	Y	W	-20.3
SGB1-71	E	I	Y	T	K	Y	H	E	Y	K	Q	M	T	I	T	-17.3
SGB1-72	Q	M	Q	N	K	E	H	M	A	K	E	M	I	I	W	-8.0

Table S11: Sixth generation of anti-RBD SGB1. All values of ΔG are kilocalories per mole.

Code \ Resid	2	4	6	8	13	15	17	19	42	44	46	49	51	53	55	ΔG
SGB1-25	S	N	S	H	L	W	N	S	P	N	P	Q	L	V	I	-62.2
FabRBD	X	X	X	X	X	X	X	X	X	X	X	X	X	X	X	-50.2
SGB1-49	Q	Q	Q	R	E	A	L	L	R	Q	M	S	I	Y	W	-46.5
SGB1-26	I	M	E	N	M	L	Y	I	N	L	R	I	A	R	M	-46.0
SGB1-27	Y	L	W	M	E	A	L	L	A	T	E	M	T	I	T	-45.6
SGB1-61	I	Q	E	R	H	Y	T	E	Y	T	Q	S	T	Y	T	-43.2
SGB1-28	E	I	Y	T	K	Y	H	E	L	I	W	K	S	E	L	-41.7
SGB1-37	Q	Q	Q	R	H	E	T	M	R	Q	M	N	Y	H	N	-41.3
SGB1-62	Y	L	W	M	H	E	T	M	Y	K	Q	S	I	Y	W	-41.2
SGB1-50	Y	L	W	M	H	E	T	M	Y	K	Q	N	Y	H	N	-41.0
SGB1-63	Y	L	W	M	E	A	L	L	N	L	R	I	A	R	M	-38.8
SGB1-64	Y	L	W	M	E	A	L	L	Y	K	Q	N	Y	H	N	-38.2
SGB1-65	Q	Q	Q	R	E	A	L	L	P	N	P	Q	L	V	I	-36.7

Table S12: Sixth generation children. All values of ΔG are kilocalories per mole.

Code \ Resid	2	4	6	8	13	15	17	19	42	44	46	49	51	53	55	ΔG
SGB1-73	Y	Q	W	R	H	E	T	M	Y	Q	Q	S	Y	Y	N	-49.2
SGB1-74	I	Q	E	R	H	Y	T	E	L	I	W	K	S	E	L	-38.2
SGB1-75	Y	L	W	M	E	A	L	L	P	N	P	Q	L	V	I	-30.0
SGB1-76	Y	L	W	M	E	A	L	L	Y	K	Q	I	A	R	M	-26.5
SGB1-77	Q	L	Q	M	H	E	T	M	R	K	M	N	I	H	W	-26.4
SGB1-78	S	N	S	H	E	A	L	L	P	N	P	S	I	Y	W	-26.2
SGB1-79	Y	L	W	M	M	L	Y	I	A	T	E	I	A	R	M	-19.4
SGB1-80	E	I	Y	T	K	Y	H	E	Y	T	Q	S	T	Y	T	-17.6
SGB1-81	Q	Q	Q	R	E	A	L	L	Y	K	Q	N	Y	H	N	-17.4
SGB1-82	Y	L	W	M	H	E	T	M	N	L	R	N	Y	H	N	-10.4
SGB1-83	I	M	E	N	E	A	L	L	N	L	R	M	T	I	T	-3.8
SGB1-84	Q	Q	Q	R	L	W	N	S	R	Q	M	Q	L	V	I	0.1

Table S13: Seventh generation of anti-RBD SGB1. All values of ΔG are kilocalories per mole.

Code \ Resid	2	4	6	8	13	15	17	19	42	44	46	49	51	53	55	ΔG
SGB1-25	S	N	S	H	L	W	N	S	P	N	P	Q	L	V	I	-62.2
FabRBD	X	X	X	X	X	X	X	X	X	X	X	X	X	X	X	-50.2
SGB1-73	Y	Q	W	R	H	E	T	M	Y	Q	Q	S	Y	Y	N	-49.2
SGB1-49	Q	Q	Q	R	E	A	L	L	R	Q	M	S	I	Y	W	-46.5
SGB1-26	I	M	E	N	M	L	Y	I	N	L	R	I	A	R	M	-46.0
SGB1-27	Y	L	W	M	E	A	L	L	A	T	E	M	T	I	T	-45.6
SGB1-61	I	Q	E	R	H	Y	T	E	Y	T	Q	S	T	Y	T	-43.2
SGB1-28	E	I	Y	T	K	Y	H	E	L	I	W	K	S	E	L	-41.7
SGB1-37	Q	Q	Q	R	H	E	T	M	R	Q	M	N	Y	H	N	-41.3
SGB1-62	Y	L	W	M	H	E	T	M	Y	K	Q	S	I	Y	W	-41.2
SGB1-50	Y	L	W	M	H	E	T	M	Y	K	Q	N	Y	H	N	-41.0
SGB1-63	Y	L	W	M	E	A	L	L	N	L	R	I	A	R	M	-38.8
SGB1-74	I	Q	E	R	H	Y	T	E	L	I	W	K	S	E	L	-38.2

Table S14: Seventh generation children. All values of ΔG are kilocalories per mole.

Code \ Resid	2	4	6	8	13	15	17	19	42	44	46	49	51	53	55	ΔG
SGB1-85	Y	Q	W	R	L	W	N	S	Y	Q	Q	Q	L	V	I	-56.7
SGB1-86	E	Q	Y	R	H	Y	T	E	L	Q	W	K	Y	E	N	-45.7
SGB1-87	I	M	E	N	E	A	L	L	N	L	R	S	I	Y	W	-42.3
SGB1-88	Q	Q	Q	R	M	L	Y	I	R	Q	M	I	A	R	M	-31.7
SGB1-89	Y	L	W	M	H	E	T	M	Y	K	Q	S	I	H	W	-28.7
SGB1-89	I	Q	E	R	E	A	L	L	Y	T	Q	M	T	I	T	-28.2
SGB1-91	Y	L	W	M	H	Y	T	E	A	T	E	S	T	Y	T	-21.9
SGB1-92	S	N	S	H	H	E	T	M	P	N	P	S	Y	Y	N	-21.1
SGB1-93	Y	Q	W	R	H	A	T	L	N	I	R	I	S	R	L	-22.0
SGB1-94	Q	I	Q	T	K	E	H	M	R	I	M	N	S	H	L	-20.9
SGB1-95	I	L	E	M	E	Y	L	E	L	L	W	K	A	E	M	-11.4
SGB1-96	Y	L	W	M	H	E	T	M	Y	K	Q	N	Y	Y	N	-7.0

Table S15: Eighth generation of anti-RBD SGB1. All values of ΔG are kilocalories per mole.

Code \ Resid	2	4	6	8	13	15	17	19	42	44	46	49	51	53	55	ΔG
SGB1-25	S	N	S	H	L	W	N	S	P	N	P	Q	L	V	I	-62.2
SGB1-85	Y	Q	W	R	L	W	N	S	Y	Q	Q	Q	L	V	I	-56.7
FabRBD	X	X	X	X	X	X	X	X	X	X	X	X	X	X	X	-50.2
SGB1-73	Y	Q	W	R	H	E	T	M	Y	Q	Q	S	Y	Y	N	-49.2
SGB1-49	Q	Q	Q	R	E	A	L	L	R	Q	M	S	I	Y	W	-46.5
SGB1-26	I	M	E	N	M	L	Y	I	N	L	R	I	A	R	M	-46.0
SGB1-86	E	Q	Y	R	H	Y	T	E	L	Q	W	K	Y	E	N	-45.7
SGB1-27	Y	L	W	M	E	A	L	L	A	T	E	M	T	I	T	-45.6
SGB1-61	I	Q	E	R	H	Y	T	E	Y	T	Q	S	T	Y	T	-43.2
SGB1-87	I	M	E	N	E	A	L	L	N	L	R	S	I	Y	W	-42.3
SGB1-28	E	I	Y	T	K	Y	H	E	L	I	W	K	S	E	L	-41.7
SGB1-37	Q	Q	Q	R	H	E	T	M	R	Q	M	N	Y	H	N	-41.3
SGB1-62	Y	L	W	M	H	E	T	M	Y	K	Q	S	I	Y	W	-41.2

Table S16: Eighth generation children. All values of ΔG are kilocalories per mole.

Code \ Resid	2	4	6	8	13	15	17	19	42	44	46	49	51	53	55	ΔG
SGB1-97	I	L	E	M	E	Y	L	E	Y	T	Q	S	T	Y	T	-44.3
SGB1-98	I	M	E	N	M	L	Y	I	L	Q	W	K	Y	E	N	-37.3
SGB1-99	S	N	S	H	L	W	N	S	Y	Q	Q	Q	L	V	I	-35.8
SGB1-100	Y	Q	W	R	H	A	T	L	A	T	E	M	T	I	T	-26.4
SGB1-101	Y	Q	W	R	L	W	N	S	P	N	P	Q	L	V	I	-24.5
SGB1-102	I	M	E	N	E	A	L	L	L	I	W	K	S	E	L	-23.4
SGB1-103	Y	Q	W	R	H	E	T	M	R	Q	M	S	I	Y	W	-22.6
SGB1-104	Q	Q	Q	R	E	A	L	L	Y	Q	Q	S	Y	Y	N	-21.4
SGB1-105	E	Q	Y	R	H	Y	T	E	N	L	R	I	A	R	M	-17.5
SGB1-106	Y	Q	W	R	H	E	T	M	R	Q	Q	S	Y	Y	N	-12.9
SGB1-107	Q	L	Q	M	H	E	T	M	Y	K	M	N	I	H	W	-12.2
SGB1-108	E	I	Y	T	K	Y	H	E	N	L	R	S	I	Y	W	-10.9

Table S17: Ninth generation of anti-RBD SGB1. All values of ΔG are kilocalories per mole.

Code \ Resid	2	4	6	8	13	15	17	19	42	44	46	49	51	53	55	ΔG
SGB1-25	S	N	S	H	L	W	N	S	P	N	P	Q	L	V	I	-62.2
SGB1-85	Y	Q	W	R	L	W	N	S	Y	Q	Q	Q	L	V	I	-56.7
FabRBD	X	X	X	X	X	X	X	X	X	X	X	X	X	X	X	-50.2
SGB1-73	Y	Q	W	R	H	E	T	M	Y	Q	Q	S	Y	Y	N	-49.2
SGB1-49	Q	Q	Q	R	E	A	L	L	R	Q	M	S	I	Y	W	-46.5
SGB1-26	I	M	E	N	M	L	Y	I	N	L	R	I	A	R	M	-46.0
SGB1-86	E	Q	Y	R	H	Y	T	E	L	Q	W	K	Y	E	N	-45.7
SGB1-27	Y	L	W	M	E	A	L	L	A	T	E	M	T	I	T	-45.6
SGB1-97	I	L	E	M	E	Y	L	E	Y	T	Q	S	T	Y	T	-44.3
SGB1-61	I	Q	E	R	H	Y	T	E	Y	T	Q	S	T	Y	T	-43.2
SGB1-87	I	M	E	N	E	A	L	L	N	L	R	S	I	Y	W	-42.3
SGB1-28	E	I	Y	T	K	Y	H	E	L	I	W	K	S	E	L	-41.7
SGB1-37	Q	Q	Q	R	H	E	T	M	R	Q	M	N	Y	H	N	-41.3

Table S18: Ninth generation children. All values of ΔG are kilocalories per mole.

Code \ Resid	2	4	6	8	13	15	17	19	42	44	46	49	51	53	55	ΔG
SGB1-109	S	Q	S	R	L	W	N	S	P	Q	P	Q	L	V	I	-74.1
SGB1-110	E	Q	Y	R	M	L	Y	I	L	Q	W	I	A	R	M	-50.5
SGB1-111	I	M	E	N	H	Y	T	E	N	L	R	K	Y	E	N	-40.7
SGB1-112	E	Q	Y	R	H	Y	T	E	R	Q	W	K	Y	E	N	-37.4
SGB1-113	I	M	E	N	E	A	L	L	Y	T	Q	S	T	Y	T	-28.2
SGB1-114	I	L	E	M	E	A	L	L	Y	T	Q	M	T	I	T	-27.5
SGB1-115	Y	N	W	H	L	W	N	S	Y	N	Q	Q	L	V	I	-27.2
SGB1-116	Y	Q	W	R	E	A	L	L	Y	Q	Q	S	I	Y	W	-26.0
SGB1-117	Q	Q	Q	R	H	E	T	M	R	Q	M	S	Y	Y	N	-23.8
SGB1-118	I	Q	E	R	H	Y	T	E	N	L	R	S	I	Y	W	-14.9
SGB1-119	Q	I	Q	T	K	E	H	M	L	I	M	N	S	H	L	-11.2
SGB1-120	Y	L	W	M	E	Y	L	E	A	T	E	S	T	Y	T	-9.1

Table S19: Tenth generation of anti-RBD SGB1. All values of ΔG are kilocalories per mole.

Code \ Resid	2	4	6	8	13	15	17	19	42	44	46	49	51	53	55	ΔG
SGB1-109	S	Q	S	R	L	W	N	S	P	Q	P	Q	L	V	I	-74.1
SGB1-25	S	N	S	H	L	W	N	S	P	N	P	Q	L	V	I	-62.2
SGB1-85	Y	Q	W	R	L	W	N	S	Y	Q	Q	Q	L	V	I	-56.7
SGB1-110	E	Q	Y	R	M	L	Y	I	L	Q	W	I	A	R	M	-50.5
FabRBD	X	X	X	X	X	X	X	X	X	X	X	X	X	X	X	-50.2
SGB1-73	Y	Q	W	R	H	E	T	M	Y	Q	Q	S	Y	Y	N	-49.2
SGB1-49	Q	Q	Q	R	E	A	L	L	R	Q	M	S	I	Y	W	-46.5
SGB1-26	I	M	E	N	M	L	Y	I	N	L	R	I	A	R	M	-46.0
SGB1-86	E	Q	Y	R	H	Y	T	E	L	Q	W	K	Y	E	N	-45.7
SGB1-27	Y	L	W	M	E	A	L	L	A	T	E	M	T	I	T	-45.6
SGB1-97	I	L	E	M	E	Y	L	E	Y	T	Q	S	T	Y	T	-44.3
SGB1-61	I	Q	E	R	H	Y	T	E	Y	T	Q	S	T	Y	T	-43.2
SGB1-87	I	M	E	N	E	A	L	L	N	L	R	S	I	Y	W	-42.3

Table S20: Tenth generation children. All values of ΔG are kilocalories per mole.

Code \ Resid	2	4	6	8	13	15	17	19	42	44	46	49	51	53	55	ΔG
SGB1-121	I	Q	E	R	E	A	L	L	Y	T	Q	S	I	Y	W	-75.6
SGB1-122	Y	Q	W	R	E	E	L	M	Y	Q	Q	S	I	Y	W	-68.5
SGB1-123	Q	Q	Q	R	H	A	T	L	R	Q	M	S	Y	Y	N	-49.4
SGB1-124	I	L	E	M	E	Y	L	E	A	T	Q	S	T	Y	T	-34.6
SGB1-125	E	Q	Y	R	M	L	Y	I	Y	Q	Q	Q	L	V	I	-32.8
SGB1-126	Y	L	W	M	E	A	L	L	Y	T	E	M	T	I	T	-28.8
SGB1-127	Y	Q	W	R	L	W	N	S	L	Q	W	I	A	R	M	-23.9
SGB1-128	S	Q	S	R	L	W	N	S	P	N	P	Q	L	V	I	-22.7
SGB1-129	I	M	E	N	H	Y	T	E	N	L	R	S	T	Y	T	-22.5
SGB1-130	S	N	S	H	L	W	N	S	P	Q	P	Q	L	V	I	-14.6
SGB1-131	E	M	Y	N	M	Y	Y	E	L	L	W	K	A	E	M	-21.5
SGB1-132	I	Q	E	R	H	L	T	I	N	Q	R	I	Y	R	N	-3.8

Table S21: Eleventh generation of anti-RBD SGB1. All values of ΔG are kilocalories per mole.

Code \ Resid	2	4	6	8	13	15	17	19	42	44	46	49	51	53	55	ΔG
SGB1-121	I	Q	E	R	E	A	L	L	Y	T	Q	S	I	Y	W	-75.6
SGB1-109	S	Q	S	R	L	W	N	S	P	Q	P	Q	L	V	I	-74.1
SGB1-122	Y	Q	W	R	E	E	L	M	Y	Q	Q	S	I	Y	W	-68.5
SGB1-25	S	N	S	H	L	W	N	S	P	N	P	Q	L	V	I	-62.2
SGB1-85	Y	Q	W	R	L	W	N	S	Y	Q	Q	Q	L	V	I	-56.7
SGB1-110	E	Q	Y	R	M	L	Y	I	L	Q	W	I	A	R	M	-50.5
FabRBD	X	X	X	X	X	X	X	X	X	X	X	X	X	X	X	-50.2
SGB1-123	Q	Q	Q	R	H	A	T	L	R	Q	M	S	Y	Y	N	-49.4
SGB1-73	Y	Q	W	R	H	E	T	M	Y	Q	Q	S	Y	Y	N	-49.2
SGB1-49	Q	Q	Q	R	E	A	L	L	R	Q	M	S	I	Y	W	-46.5
SGB1-26	I	M	E	N	M	L	Y	I	N	L	R	I	A	R	M	-46.0
SGB1-86	E	Q	Y	R	H	Y	T	E	L	Q	W	K	Y	E	N	-45.7
SGB1-27	Y	L	W	M	E	A	L	L	A	T	E	M	T	I	T	-45.6

Table S22: Eleventh generation children. All values of ΔG are kilocalories per mole.

Code \ Resid	2	4	6	8	13	15	17	19	42	44	46	49	51	53	55	ΔG
SGB1-133	Y	Q	W	R	M	W	Y	S	Y	Q	Q	Q	A	V	M	-54.1
SGB1-134	Q	M	Q	N	M	A	Y	L	N	L	M	S	A	Y	M	-48.7
SGB1-135	Y	Q	E	R	E	A	L	L	Y	T	Q	S	I	Y	W	-48.2
SGB1-136	E	Q	Y	R	E	A	L	L	L	Q	W	M	T	I	T	-34.9
SGB1-137	Y	Q	Q	R	H	A	T	L	Y	Q	Q	S	Y	Y	N	-33.5
SGB1-138	I	Q	W	R	E	E	L	M	Y	Q	Q	S	I	Y	W	-29.6
SGB1-139	S	Q	S	R	L	W	N	S	A	Q	A	Q	L	V	I	-26.0
SGB1-140	Y	L	W	M	H	Y	T	E	A	T	E	K	Y	E	N	-25.2
SGB1-141	E	Q	Y	R	L	L	N	I	L	Q	W	I	L	R	I	-22.5
SGB1-142	Q	Q	W	R	H	E	T	M	R	Q	M	S	Y	Y	N	-22.2
SGB1-143	S	N	S	H	L	W	N	S	A	N	A	Q	L	V	I	-16.6
SGB1-144	I	Q	E	R	E	L	L	I	R	Q	R	I	I	R	W	-14.7

Table S23: Twelfth generation of anti-RBD SGB1. All values of ΔG are kilocalories per mole.

Code \ Resid	2	4	6	8	13	15	17	19	42	44	46	49	51	53	55	ΔG
SGB1-121	I	Q	E	R	E	A	L	L	Y	T	Q	S	I	Y	W	-75.6
SGB1-109	S	Q	S	R	L	W	N	S	P	Q	P	Q	L	V	I	-74.1
SGB1-122	Y	Q	W	R	E	E	L	M	Y	Q	Q	S	I	Y	W	-68.5
SGB1-25	S	N	S	H	L	W	N	S	P	N	P	Q	L	V	I	-62.2
SGB1-85	Y	Q	W	R	L	W	N	S	Y	Q	Q	Q	L	V	I	-56.7
SGB1-133	Y	Q	W	R	M	W	Y	S	Y	Q	Q	Q	A	V	M	-54.1
SGB1-110	E	Q	Y	R	M	L	Y	I	L	Q	W	I	A	R	M	-50.5
FabRBD	X	X	X	X	X	X	X	X	X	X	X	X	X	X	X	-50.2
SGB1-123	Q	Q	Q	R	H	A	T	L	R	Q	M	S	Y	Y	N	-49.4
SGB1-73	Y	Q	W	R	H	E	T	M	Y	Q	Q	S	Y	Y	N	-49.2
SGB1-134	Q	M	Q	N	M	A	Y	L	N	L	M	S	A	Y	M	-48.7
SGB1-135	Y	Q	E	R	E	A	L	L	Y	T	Q	S	I	Y	W	-48.2
SGB1-49	Q	Q	Q	R	E	A	L	L	R	Q	M	S	I	Y	W	-46.5

Table S24: Twelfth generation children. All values of ΔG are kilocalories per mole.

Code \ Resid	2	4	6	8	13	15	17	19	42	44	46	49	51	53	55	ΔG
SGB1-145	Y	Q	E	R	E	A	L	L	Y	Q	Q	S	I	Y	W	-62.7
SGB1-146	Y	Q	W	R	E	E	L	L	Y	T	Q	S	I	Y	W	-44.7
SGB1-147	I	Q	E	R	E	A	L	M	Y	Q	Q	S	I	Y	W	-36.0
SGB1-148	Q	M	Q	N	H	E	T	M	N	L	M	S	Y	Y	N	-34.8
SGB1-149	Y	Q	W	R	M	W	Y	S	Y	Q	Q	Q	L	V	M	-29.1
SGB1-150	Q	Q	Q	R	E	A	L	L	R	T	M	S	I	Y	W	-27.9
SGB1-151	Q	Q	Q	R	M	A	Y	L	R	Q	M	S	A	Y	M	-26.0
SGB1-152	I	M	E	N	M	L	Y	I	Y	Q	Q	S	I	Y	W	-25.2
SGB1-153	Y	Q	W	R	L	W	N	S	Y	Q	Q	Q	A	V	I	-22.4
SGB1-154	Y	Q	W	R	M	A	Y	L	Y	Q	Q	S	A	Y	M	-21.4
SGB1-155	I	Q	E	R	E	E	L	M	N	L	R	I	A	R	M	-18.0
SGB1-156	E	Q	Y	R	H	L	T	I	L	Q	W	I	Y	R	N	-14.9

Table S25: Thirteenth generation of anti-RBD SGB1. All values of ΔG are kilocalories per mole.

Code \ Resid	2	4	6	8	13	15	17	19	42	44	46	49	51	53	55	ΔG
SGB1-121	I	Q	E	R	E	A	L	L	Y	T	Q	S	I	Y	W	-75.6
SGB1-109	S	Q	S	R	L	W	N	S	P	Q	P	Q	L	V	I	-74.1
SGB1-122	Y	Q	W	R	E	E	L	M	Y	Q	Q	S	I	Y	W	-68.5
SGB1-145	Y	Q	E	R	E	A	L	L	Y	Q	Q	S	I	Y	W	-62.7
SGB1-25	S	N	S	H	L	W	N	S	P	N	P	Q	L	V	I	-62.2
SGB1-85	Y	Q	W	R	L	W	N	S	Y	Q	Q	Q	L	V	I	-56.7
SGB1-133	Y	Q	W	R	M	W	Y	S	Y	Q	Q	Q	A	V	M	-54.1
SGB1-110	E	Q	Y	R	M	L	Y	I	L	Q	W	I	A	R	M	-50.5
FabRBD	X	X	X	X	X	X	X	X	X	X	X	X	X	X	X	-50.2
SGB1-123	Q	Q	Q	R	H	A	T	L	R	Q	M	S	Y	Y	N	-49.4
SGB1-73	Y	Q	W	R	H	E	T	M	Y	Q	Q	S	Y	Y	N	-49.2
SGB1-134	Q	M	Q	N	M	A	Y	L	N	L	M	S	A	Y	M	-48.7
SGB1-135	Y	Q	E	R	E	A	L	L	Y	T	Q	S	I	Y	W	-48.2

Table S26: Thirteenth generation children. All values of ΔG are kilocalories per mole.

Code \ Resid	2	4	6	8	13	15	17	19	42	44	46	49	51	53	55	ΔG
SGB1-157	Y	Q	E	R	E	A	L	L	Y	Q	Q	Q	L	V	I	-64.7
SGB1-158	Y	Q	W	R	M	W	Y	S	L	Q	W	I	A	R	M	-45.6
SGB1-159	E	Q	Y	R	M	L	Y	I	Y	Q	Q	Q	A	V	M	-44.2
SGB1-160	Y	Q	E	R	E	A	L	L	N	L	M	S	A	Y	M	-41.0
SGB1-161	Y	Q	W	R	L	W	N	S	Y	Q	Q	S	I	Y	W	-36.8
SGB1-162	Y	Q	W	R	E	E	L	M	Y	T	Q	S	L	Y	W	-36.6
SGB1-163	I	Q	E	R	E	A	L	L	Y	Q	Q	S	I	Y	W	-35.4
SGB1-164	Y	Q	W	R	H	E	T	L	Y	Q	Q	S	Y	Y	N	-31.1
SGB1-165	Q	M	Q	N	M	A	Y	L	Y	T	Q	S	I	Y	W	-23.5
SGB1-166	I	Q	E	R	E	L	L	I	R	L	R	I	I	R	W	-20.4
SGB1-167	Q	M	Q	N	M	A	Y	L	N	Q	M	S	A	Y	M	-14.7
SGB1-168	Q	Q	Q	R	H	A	T	M	R	Q	M	S	Y	Y	N	-14.1

Table S27: Fourteenth generation of anti-RBD SGB1. All values of ΔG are kilocalories per mole.

Code \ Resid	2	4	6	8	13	15	17	19	42	44	46	49	51	53	55	ΔG
SGB1-121	I	Q	E	R	E	A	L	L	Y	T	Q	S	I	Y	W	-75.6
SGB1-109	S	Q	S	R	L	W	N	S	P	Q	P	Q	L	V	I	-74.1
SGB1-122	Y	Q	W	R	E	E	L	M	Y	Q	Q	S	I	Y	W	-68.5
SGB1-157	Y	Q	E	R	E	A	L	L	Y	Q	Q	Q	L	V	I	-64.7
SGB1-145	Y	Q	E	R	E	A	L	L	Y	Q	Q	S	I	Y	W	-62.7
SGB1-25	S	N	S	H	L	W	N	S	P	N	P	Q	L	V	I	-62.2
SGB1-85	Y	Q	W	R	L	W	N	S	Y	Q	Q	Q	L	V	I	-56.7
SGB1-133	Y	Q	W	R	M	W	Y	S	Y	Q	Q	Q	A	V	M	-54.1
SGB1-110	E	Q	Y	R	M	L	Y	I	L	Q	W	I	A	R	M	-50.5
FabRBD	X	X	X	X	X	X	X	X	X	X	X	X	X	X	X	-50.23
SGB1-123	Q	Q	Q	R	H	A	T	L	R	Q	M	S	Y	Y	N	-49.4
SGB1-73	Y	Q	W	R	H	E	T	M	Y	Q	Q	S	Y	Y	N	-49.2
SGB1-134	Q	M	Q	N	M	A	Y	L	N	L	M	S	A	Y	M	-48.7

Table S28: Fourteenth generation children. All values of ΔG are kilocalories per mole.

Code \ Resid	2	4	6	8	13	15	17	19	42	44	46	49	51	53	55	ΔG
SGB1-169	Y	Q	W	R	M	W	N	S	Y	Q	Q	Q	A	V	M	-55.0
SGB1-170	Y	Q	E	R	E	A	L	L	R	Q	Q	S	I	Y	W	-51.2
SGB1-171	Y	Q	W	R	E	E	L	M	Y	Q	Q	S	L	Y	W	-44.5
SGB1-172	Y	Q	W	R	L	W	Y	S	Y	Q	Q	Q	L	V	I	-38.7
SGB1-173	Y	Q	E	R	E	A	L	L	Y	Q	Q	Q	I	V	W	-36.7
SGB1-174	Q	Q	Q	R	M	A	Y	L	L	Q	M	S	A	Y	M	-36.2
SGB1-175	Q	Q	Q	R	H	A	T	L	N	Q	M	S	Y	Y	N	-36.2
SGB1-176	Y	Q	E	R	E	A	L	L	Y	Q	Q	S	L	Y	I	-34.4
SGB1-177	Q	Q	Q	R	E	A	L	L	Y	T	M	S	I	Y	W	-33.6
SGB1-178	I	Q	E	R	E	A	L	L	Y	T	Q	S	L	Y	W	-31.9
SGB1-179	Y	M	W	N	M	E	Y	M	Y	L	Q	S	A	Y	M	-24.7
SGB1-180	E	Q	Y	R	H	L	T	I	R	Q	W	I	Y	R	N	-8.7

Table S29: Fifteenth generation of anti-RBD SGB1. All values of ΔG are kilocalories per mole.

Code \ Resid	2	4	6	8	13	15	17	19	42	44	46	49	51	53	55	ΔG
SGB1-121	I	Q	E	R	E	A	L	L	Y	T	Q	S	I	Y	W	-75.6
SGB1-109	S	Q	S	R	L	W	N	S	P	Q	P	Q	L	V	I	-74.1
SGB1-122	Y	Q	W	R	E	E	L	M	Y	Q	Q	S	I	Y	W	-68.5
SGB1-157	Y	Q	E	R	E	A	L	L	Y	Q	Q	Q	L	V	I	-64.7
SGB1-145	Y	Q	E	R	E	A	L	L	Y	Q	Q	S	I	Y	W	-62.7
SGB1-25	S	N	S	H	L	W	N	S	P	N	P	Q	L	V	I	-62.2
SGB1-85	Y	Q	W	R	L	W	N	S	Y	Q	Q	Q	L	V	I	-56.7
SGB1-169	Y	Q	W	R	M	W	N	S	Y	Q	Q	Q	A	V	M	-55.0
SGB1-133	Y	Q	W	R	M	W	Y	S	Y	Q	Q	Q	A	V	M	-54.1
SGB1-170	Y	Q	E	R	E	A	L	L	R	Q	Q	S	I	Y	W	-51.2
SGB1-110	E	Q	Y	R	M	L	Y	I	L	Q	W	I	A	R	M	-50.5
FabRBD	X	X	X	X	X	X	X	X	X	X	X	X	X	X	X	-50.2
SGB1-123	Q	Q	Q	R	H	A	T	L	R	Q	M	S	Y	Y	N	-49.4

Table S30: Fifteenth generation children. All values of ΔG are kilocalories per mole.

Code \ Resid	2	4	6	8	13	15	17	19	42	44	46	49	51	53	55	ΔG
SGB1-181	Y	Q	E	R	M	W	N	S	Y	Q	Q	Q	A	V	M	-52.9
SGB1-182	Y	Q	E	R	E	A	L	L	Y	Q	Q	Q	L	Y	I	-51.1
SGB1-183	Y	Q	E	R	E	A	L	L	Y	Q	Q	Q	A	V	M	-49.9
SGB1-184	I	Q	D	R	D	A	L	L	Y	Q	Q	S	I	Y	W	-49.5
SGB1-185	Q	Q	Q	R	M	A	Y	L	L	Q	W	S	A	Y	M	-32.5
SGB1-186	Y	Q	E	R	E	A	L	L	Y	Q	Q	S	I	V	W	-30.3
SGB1-187	Y	Q	W	R	M	W	Y	S	R	Q	Q	S	I	Y	W	-29.1
SGB1-188	Q	Q	Q	R	H	A	T	L	Y	Q	M	S	Y	Y	N	-27.8
SGB1-189	I	Q	D	R	D	A	L	L	Y	T	Q	S	I	Y	W	-21.4
SGB1-190	Y	M	W	N	M	E	Y	M	N	L	Q	S	A	Y	M	-21.0
SGB1-191	Y	Q	W	R	L	W	N	S	Y	Q	Q	Q	L	V	I	-20.8
SGB1-192	E	Q	Y	R	H	L	T	I	R	Q	M	I	Y	R	N	-19.8

Table S31: Sixteenth generation of anti-RBD SGB1. All values of ΔG are kilocalories per mole.

Code \ Resid	2	4	6	8	13	15	17	19	42	44	46	49	51	53	55	ΔG
SGB1-121	I	Q	E	R	E	A	L	L	Y	T	Q	S	I	Y	W	-75.6
SGB1-109	S	Q	S	R	L	W	N	S	P	Q	P	Q	L	V	I	-74.1
SGB1-122	Y	Q	W	R	E	E	L	M	Y	Q	Q	S	I	Y	W	-68.5
SGB1-157	Y	Q	E	R	E	A	L	L	Y	Q	Q	Q	L	V	I	-64.7
SGB1-145	Y	Q	E	R	E	A	L	L	Y	Q	Q	S	I	Y	W	-62.7
SGB1-25	S	N	S	H	L	W	N	S	P	N	P	Q	L	V	I	-62.2
SGB1-85	Y	Q	W	R	L	W	N	S	Y	Q	Q	Q	L	V	I	-56.7
SGB1-169	Y	Q	W	R	M	W	N	S	Y	Q	Q	Q	A	V	M	-55.0
SGB1-133	Y	Q	W	R	M	W	Y	S	Y	Q	Q	Q	A	V	M	-54.0
SGB1-181	Y	Q	E	R	M	W	N	S	Y	Q	Q	Q	A	V	M	-53.0
SGB1-170	Y	Q	E	R	E	A	L	L	R	Q	Q	S	I	Y	W	-51.2
SGB1-182	Y	Q	E	R	E	A	L	L	Y	Q	Q	Q	L	Y	I	-51.1
SGB1-110	E	Q	Y	R	M	L	Y	I	L	Q	W	I	A	R	M	-50.5
FabRBD	X	X	X	X	X	X	X	X	X	X	X	X	X	X	X	-50.2
GB1-REF	T	K	I	N	K	E	T	E	E	T	D	T	T	T	T	-19.5

S3 ΔG_{bind} per unit of contact area analysis

In this study, we used the VMD software (Visual Molecular Dynamics) to calculate the contact area between antibodies and the RBD antigen. The objective was to estimate the ΔG_{bind} per unit of contact area, exploring the use of this metric as a convergence criterion in future studies. The following commands were used in the Tcl terminal of VMD to perform the analysis of the B38 antibody bound to RBD:

```
set molecule1 [atomselect top "resid 1 to 185"]
set molecule2 [atomselect top "resid 186 to 622"]
set complex [atomselect top "resid 1 to 622"]
set area_molecule1 [measure sasa 1.4 $molecule1]
set area_molecule2 [measure sasa 1.4 $molecule2]
set area_complex [measure sasa 1.4 $complex]
set contact_area [expr ($area_molecule1 + $area_molecule2) - $area_complex]
```

The results obtained were:

- area molecule1 (`resid 1-185`): 10184.68 Å^2
- area molecule2 (`resid 186-622`): 20254.59 Å^2
- area complex (`resid 1-622`): 27609.20 Å^2
- contact area: 2830.07 Å^2

With a binding free energy of -100.4 kcal/mol, the ΔG_{bind} per unit area was calculated as:

$$\text{G}_{\text{bind}} \text{ per unit area} = \frac{-100.4\,\text{kcal/mol}}{2830.07\,\text{Å}^2} = -0.0355\,\text{kcal/mol/Å}^2$$

This same metric was evaluated for the SGB1-121 mutant, and the following results were obtained:

- area molecule1 (`resid 1-185`): 10327.77 Å^2
- area molecule2 (`resid 186-241`): 4103.66 Å^2
- area complex (`resid 1-241`): 12678.04 Å^2
- contact area: 1753.40 Å^2

With a binding free energy of −75.6 kcal/mol, the free energy per unit area was calculated as:

$$G_{\text{bind}} \text{ per unit area} = \frac{-75.6\,\text{kcal/mol}}{1753.40\,\text{Å}^2} = -0.0431\,\text{kcal/mol/Å}^2$$

The same procedure was performed for the other SGB1 mutants, and the results can be found in Table S32:

Table S32: Sixteenth generation of anti-RBD SGB1. All values of ΔG are in kilocalories per mole, and ΔG_{area} is in kcal/mol/Å^2.

Code \ Resid	2	4	6	8	13	15	17	19	42	44	46	49	51	53	55	ΔG	ΔG_{area}
SGB1-121	I	Q	E	R	E	A	L	L	Y	T	Q	S	I	Y	W	-75.6	-0.0431
SGB1-109	S	Q	S	R	L	W	N	S	P	Q	P	Q	L	V	I	-74.1	-0.0423
SGB1-122	Y	Q	W	R	E	E	L	M	Y	Q	Q	S	I	Y	W	-68.5	-0.0391
SGB1-157	Y	Q	E	R	E	A	L	L	Y	Q	Q	Q	L	V	I	-64.7	-0.0369
SGB1-145	Y	Q	E	R	E	A	L	L	Y	Q	Q	S	I	Y	W	-62.7	-0.0358
SGB1-25	S	N	S	H	L	W	N	S	P	N	P	Q	L	V	I	-62.2	-0.0355
FabRBD	X	X	X	X	X	X	X	X	X	X	X	X	X	X	X	-50.2	-0.0355
SGB1-85	Y	Q	W	R	L	W	N	S	Y	Q	Q	Q	L	V	I	-56.7	-0.0323
SGB1-169	Y	Q	W	R	M	W	N	S	Y	Q	Q	Q	A	V	M	-55.0	-0.0314
SGB1-133	Y	Q	W	R	M	W	Y	S	Y	Q	Q	Q	A	V	M	-54.0	-0.0308
SGB1-181	Y	Q	E	R	M	W	N	S	Y	Q	Q	Q	A	V	M	-53.0	-0.0302
SGB1-170	Y	Q	E	R	E	A	L	L	R	Q	Q	S	I	Y	W	-51.2	-0.0292
SGB1-182	Y	Q	E	R	E	A	L	L	Y	Q	Q	Q	L	Y	I	-51.1	-0.0291
SGB1-110	E	Q	Y	R	M	L	Y	I	L	Q	W	I	A	R	M	-50.5	-0.0288
GB1-REF	T	K	I	N	K	E	T	E	E	T	D	T	T	T	T	-19.5	-0.0111

S4 GA Standard deviation

The GA model for RBD was designed with subsequent experimental steps in mind. Therefore, two more MDs were performed with this system, following the same methodology, as a way of evaluating the reliability of the SGB1-121 mutant model. In the first simulation (Figure S6A), a new MD of 100 ns was performed with the same SGB1-121 structure and RBD in the initial conditions of the GA (SGB1-121MD). Then, after adding a his-tag group of 6 histidines to the C-terminal region of the SGB1-121 mutant (Figure S6B), a new 100 ns MD was performed (SGB1-121$_{histag}$). This simulation is necessary because the his-tag is used in the purification step by layer chromatography on a nickel column. This comparison is crucial to assess whether the molecular recognition capacity is maintained even with the presence of the his-tag in the C-terminal region.

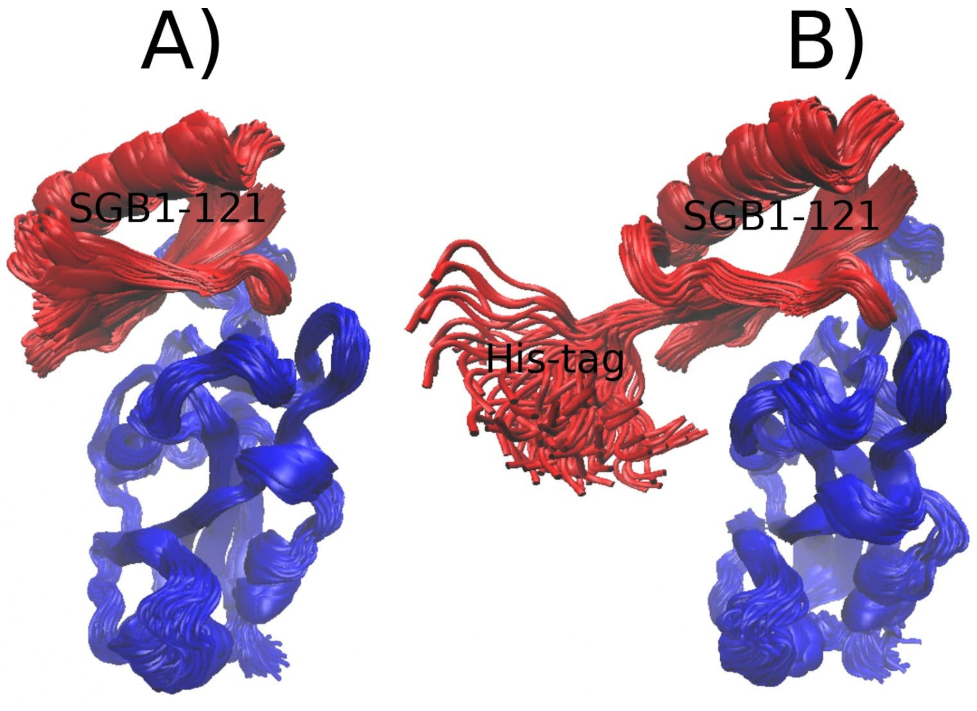

Figure S6: Illustration of the superposition of 100 ns MD trajectories for A) SGB1-121 and B) SGB1-121$_{HisTag}$ mutants.

An important point to highlight is that ΔG_{bind} is not a constant, antibodies are not covalently linked to the antigen. So if we could run a simulation long enough, we would periodically see a separation between the two. In this case, within the GA protocol it makes more sense to compare only the averages of the ΔG_{bind} within the local minimum. However, it is appropriate to show how the standard deviation follows the MA energy optimization, as we can see in Figure S7:

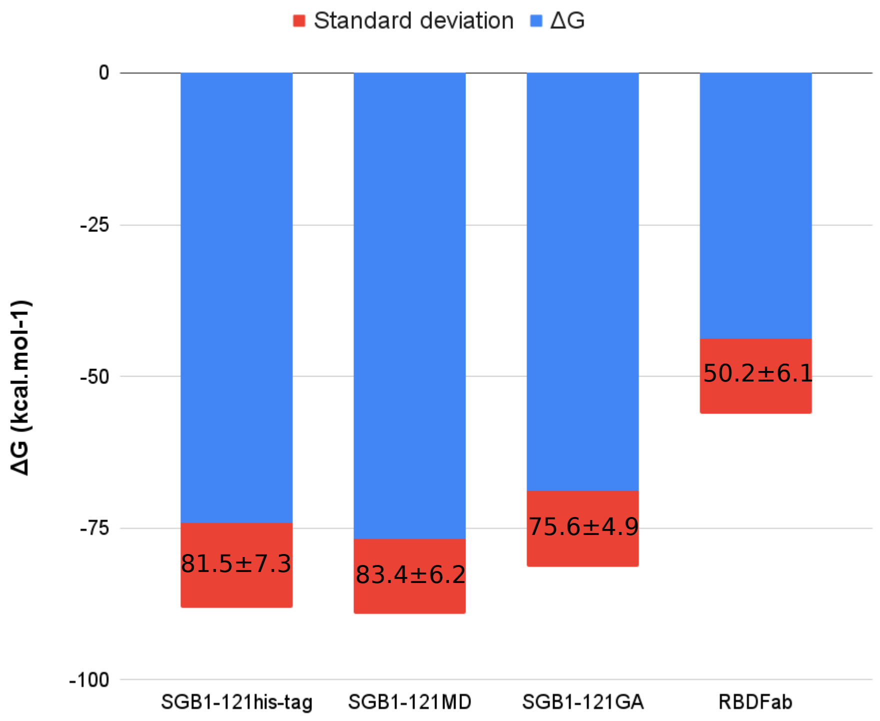

Figure S7: Standard deviation of the energy calculated for the RBD, used as a convergence criterion, and the energies of the best energy mutant SGB1-121.

Figure S7 shows the energies of -83.0±6.2 kcal.mol^{-1} for the SGB1-121MD mutant and -81.5±7.3 kcal.mol^{-1} for the SGB1-121his-tag mutant, results better than the value of -75.6±4.9 kcal.mol^{-1} obtained by SGB1-121 in GA. The energy values are very close, within the standard deviation, even in different simulations. This result confirms that the MD sampling time is sufficient to obtain reliable results.

These energy values are very far from the convergence criterion value, even considering

the standard deviation, showing that the energies were optimized by the GA in a statistically significant way. Furthermore, we noticed that even with the addition of the his-tag group in the SGB1-121$_{histag}$ mutant, there appears to have been no loss in molecular recognition capacity, an important factor to consider before committing to the experimental stage.

S5 Structural analysis

Once GA was completed, we used alphafold[4,5] software to predict the sequence structure of the best energy mutant SGB1-121$_{histag}$. This software is known in the most current literature for having high accuracy in elucidating protein structures, as can be seen in Figure S8.

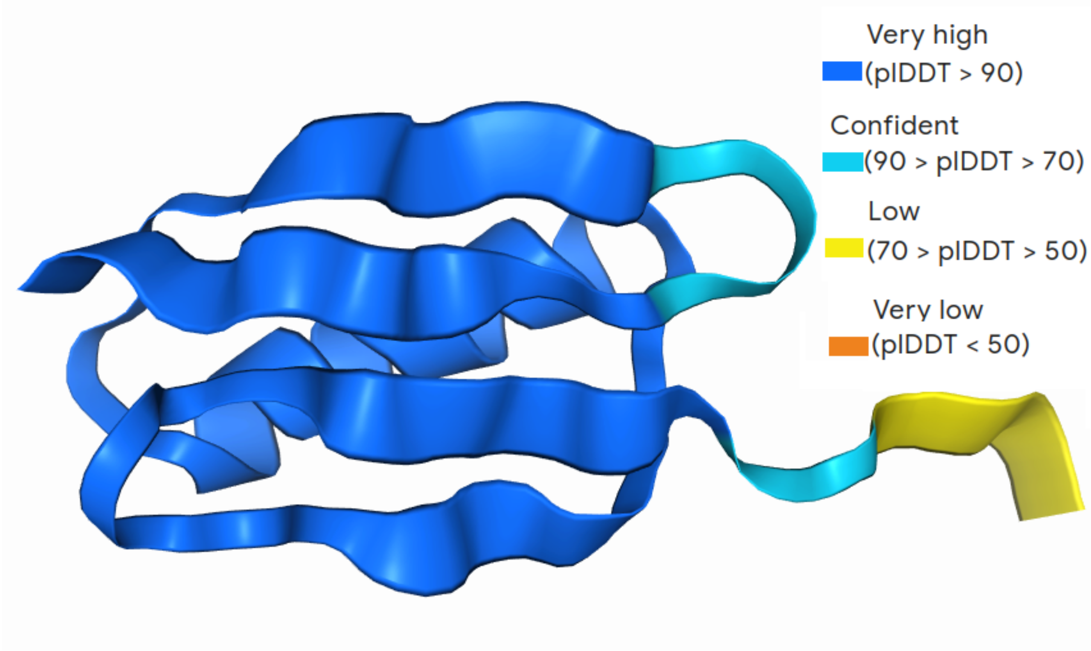

Figure S8: Illustration of the structural prediction of the SGB1-121$_{histag}$ mutant performed by alphafold. plDDT values indicate very high confidence in the predicted structure.

As can be seen in Figure S8, the alphafold predicts that the structure of the SGB1-121$_{histag}$ mutant forms the same structural motif characteristic of the original GB1, even with all residue modifications. The structure of SGB1-121$_{histag}$ remains stable forming four β-strands and an α-helix. plDDT values indicate very high confidence in the predicted structure. This prediction is very reliable and essential for the continuation of the project.

Due to the very favorable energetic contribution, we analyzed the structure of Arginine 8 in the complex formed by SGB11-121 and RBD, as seen in Figure S9:

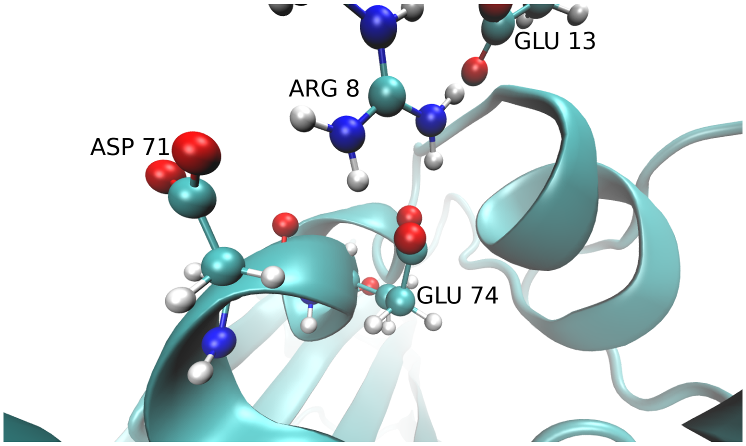

Figure S9: Individual contributions of the residues of the SGB1-121 mutant to the total ΔG_{bind}.

References

(1) Chen, J.; Li, Y.; Zhang, K.; Wang, H. Whole-Genome Sequence of Phage-Resistant Strain Escherichia coli DH5. *Genome Announcements* **2018**, *6*.

(2) Jeong, H.; Kim, H. J.; Lee, S. J. Complete Genome Sequence of Escherichia coli Strain BL21. *Genome Announcements* **2015**, *3*.

(3) Sambrook, J.; Russell, D. W. *Molecular cloning: a laboratory manual*, 4th ed.; Molecular Biology; Cold Spring Harbor Laboratory Press, 2012.

(4) Jumper, J.; Evans, R.; Pritzel, A.; Green, T.; Figurnov, M.; Ronneberger, O.; Tunyasuvunakool, K.; Bates, R.; Žídek, A.; Potapenko, A.; Bridgland, A.; Meyer, C.; Kohl, S. A. A.; Ballard, A. J.; Cowie, A.; Romera-Paredes, B.; Nikolov, S.; Jain, R.; Adler, J.; Back, T.; Petersen, S.; Reiman, D.; Clancy, E.; Zielinski, M.; Steinegger, M.; Pacholska, M.; Berghammer, T.; Bodenstein, S.; Silver, D.; Vinyals, O.; Senior, A. W.;

Kavukcuoglu, K.; Kohli, P.; Hassabis, D. Highly accurate protein structure prediction with AlphaFold. *Nature* **2021**, *596*, 583–589.

(5) Varadi, M.; Anyango, S.; Deshpande, M.; Nair, S.; Natassia, C.; Yordanova, G.; Yuan, D.; Stroe, O.; Wood, G.; Laydon, A.; Žídek, A.; Green, T.; Tunyasuvunakool, K.; Petersen, S.; Jumper, J.; Clancy, E.; Green, R.; Vora, A.; Lutfi, M.; Figurnov, M.; Cowie, A.; Hobbs, N.; Kohli, P.; Kleywegt, G.; Birney, E.; Hassabis, D.; Velankar, S. AlphaFold Protein Structure Database: massively expanding the structural coverage of protein-sequence space with high-accuracy models. *Nucleic Acids Research* **2021**, *50*, D439–D444.
